# Evolutionary and functional analysis of two‐component system in chickpea reveals CaRR13, a TypeB RR, as positive regulator of symbiosis

**DOI:** 10.1111/pbi.13649

**Published:** 2021-09-16

**Authors:** Manish Tiwari, Manisha Yadav, Baljinder Singh, Vimal Pandey, Kashif Nawaz, Sabhyata Bhatia

**Affiliations:** ^1^ National Institute of Plant Genome Research New Delhi India

**Keywords:** chickpea, nodule, cytokinin, two‐component system, evolution, histidine kinase, response regulator

## Abstract

The critical role of cytokinin in early nodulation in legumes is well known. In our study, exogenous cytokinin application to roots of the important crop legume, chickpea (*Cicer arietinum* L.), led to the formation of pseudo‐nodules even in the absence of rhizobia. Hence, a genome‐wide analysis of the cytokinin signalling, two‐component system (TCS) genes, was conducted in chickpea, *Medicago* and *Cajanus cajan*. The integrated phylogenetic, evolutionary and expression analysis of the TCS genes was carried out, which revealed that histidine kinases (HKs) were highly conserved, whereas there was diversification leading to neofunctionalization at the level of response regulators (RRs) especially the TypeB RRs. Further, the functional role of the CaHKs in nodulation was established by complementation of the *sln1Δ* mutant of yeast and *cre1* mutants of (*Medicago*) which led to restoration of the nodule‐deficient phenotype. Additionally, the highest expressing TypeB RR of chickpea, CaRR13, was functionally characterized. Its localization in the nucleus and its Y1H assay‐based interaction with the promoter of the early nodulation gene Ca*NSP2* indicated its role as a transcription factor regulating early nodulation. Overexpression, RNAi lines and complementation of *cre1* mutants with CaRR13 revealed its critical involvement as an important signalling molecule regulating early events of nodule organogenesis in chickpea.

## Introduction

Most legumes symbiotically interact with rhizobia under limiting nitrogen conditions to fix atmospheric nitrogen in specialized organs called root nodules. Symbiosis between legumes and rhizobia involves a complex molecular dialogue which is chiefly influenced by phytohormones, particularly cytokinin (Frugier *et al*., 2008). It is especially known to act as a key differentiation signal for nodule organogenesis. Exogenous cytokinin application can initiate the formation of nodule‐like structures even in the absence of rhizobia (Frugier *et al*., 2008). Ectopic cytokinin treatment leads to expression of early nodulin genes (e.g. *ENOD40, NIN, ERN1* and *NSP2*), induction of cortical cell divisions and amyloplast deposition in various legumes including *Pisum sativum, Medicago sativa, Trifolium repens, Lotus japonicus* and *Sesbania rostrata* (Frugier *et al*., 2008; Gauthier‐Coles *et al*., 2019; Murray *et al*., 2007; Tirichine *et al*., 2007). Cytokinin has been clearly shown to regulate early nodulation events such as in *Lotus japonicus*, a gain in function mutation of cytokinin receptor, histidine kinase, *LHK1*, a *CRE1* ortholog of Arabidopsis, was reported to result in spontaneous nodule formation, while loss of function of *LHK1* and Mt*CRE1* in *L. japonicus* and *Medicago truncatula,* respectively, resulted in impaired nodulation, unequivocally demonstrating that cytokinin signalling is necessary to induce cortical cell division and nodule organogenesis (Gonzalez‐Rizzo *et al*., 2006; Murray *et al*., 2007; Tirichine *et al*., 2007). In a report in *M. truncatula,* it was shown that cytokinin signalling pathway activation in epidermis and cortex was correlated with negative and positive regulation of ENOD11 expression and nodulation, respectively (Jardinaud *et al*., 2016). Another component of cytokinin signalling, the TypeB response regulators (RRs), a class of transcription factor (TF), have been reported to bind with the promoter of *NSP2* genes to activate downstream symbiotic signalling (Tan *et al*., 2020).

Cytokinin signal transduction is mediated by the two‐component system (TCS) (To and Kieber, 2008). Hence, in order to understand how cytokinin signalling occurs, it becomes imperative to identify and characterize the key elements of the TCS. The presence of TCS is reported in all forms of life such as eubacteria, archaea and eukaryotic organisms, except animalia kingdom. They are key regulatory molecules that control many biological processes, including cell division, proliferation, growth and stress (Chen *et al*., 2012; Mizuno, 2005; Pareek *et al*., 2006). TCS, also widely known as the His‐to‐Asp phosphorelay, consists of (i) a sensory receptor, that is histidine kinase, which possesses a His‐kinase (HK) domain and a receiver (Rec) domain, (ii) a phosphotransfer protein, histidine phosphotransferase (HP) and (iii) effectors, response regulators (RRs). In response to a stimulus, the kinase domain of HK auto‐phosphorylates itself at the histidine amino acid residue, which is known to be highly conserved and is required for phosphate group transfer, thereby facilitating downstream TCS signalling. Further, the signal is transferred downstream via phosphoryl transfer through HP to the aspartic acid residue of Rec domain of a TypeB RR (West and Stock, 2001). TypeB RRs function as TFs by binding with the cis‐elements in promoter regions of target genes such as *NSP2* and *bHLH*.

Histidine kinases are classified into various subclasses based on their domain organization: (i) cytokinin receptors possess CHASE (cyclase/HK‐associated sensory extracellular) domain and (ii) ethylene receptors have ethylene binding domain and phytochrome receptors have the PHY and PAS domains. Apart from these HKs, *Arabidopsis* cytokinin insensitive1 (CKI1), *Arabidopsis* histidine kinase1 (AHK1) and *Arabidopsis* histidine kinase5 (AHK5) are also annotated in the TCS family. The HPs are marked by the presence of a highly conserved xHQxKGSSxS motif that mediates phosphate transfer from Rec domain of HKs to the Rec domain of RRs. This conserved histidine is absent in some of the histidine phosphotransferase proteins, and these are considered as pseudo‐histidine phosphotransferases (PHPs) and function as negative regulators of cytokinin signalling (El‐Showk *et al*., 2013). Response regulators can be broadly categorized into TypeA, TypeB, TypeC and Pseudo RRs. TypeA and TypeC RRs contain REC and short C‐terminal extensions, and both acts as negative regulators of cytokinin signalling. TypeA RRs are induced by cytokinin treatment, while there is no effect on the expression of TypeC RRs (To and Kieber, 2008). TypeB RRs contain a REC domain at the N terminal followed by a large GARP (GOLDEN/ARR/Psr1, ˜60 amino acids motif) domain at the C terminal, which shows a distant similarity with Myb DNA binding superfamily (Mason *et al*., 2004; Tajima *et al*., 2004). Additionally, there is a diverged group of RRs which lack the conserved aspartate residues required for phosphorylation and are termed as Pseudo RRs (PRRs). A distinguishing feature of Clock PRRs is the presence of Co, Col and Toc1 motifs in the C‐terminal that regulates circadian rhythms (Nakamichi *et al*., 2010). Genes encoding TCS members have been explored at the whole‐genome level in various plant species such as Arabidopsis*, L*. *japonicus, Physcomitrella patens,* soybean, maize, rice and Chinese cabbage (Chu *et al*., 2011; Hwang *et al*., 2002; Ishida *et al*., 2009; Ishida *et al*., 2010; Lee *et al*., 2013; Liu *et al*., 2014; Schaller *et al*., 2007) These studies provide enough information about their genic and structural organization as well as their involvement in stress and developmental pathways. However, the role of cytokinin responsive TCS members and their involvement in nodulation, especially in important crop legumes such as chickpea (*Cicer arietinum* L.), still needs to be explored. Therefore, in this study, a systematic whole genome‐level identification of the genes encoding the TCS members was undertaken in three important legumes, namely chickpea (*Cicer arietinum* L.), pigeon pea (*Cajanus cajan)* and *Medicago*. Phylogenetic clustering, transcriptional regulation and evolutionary analysis of TCS genes were done in order to gain insights into their functional diversification as well as the overall divergence of legumes during the course of evolution. Further, the cytokinin responsive HKs from chickpea nodules were functionally validated for their receptor activity by complementation in yeast and *Medicago* mutants. Moreover, a chickpea TypeB RR was selected based on its expression abundance and evolutionary importance. It was functionally characterized to establish the *in planta* role during root nodulation in chickpea and legume species in general. Our understanding of the key elements of TCS family would provide insights to the cytokinin‐induced TCS signalling during nodulation which could facilitate optimized nodulation and nitrogen fixation in chickpea.

## Results

### Effect of exogenous cytokinin treatment to chickpea seedlings

**Figure 1:**
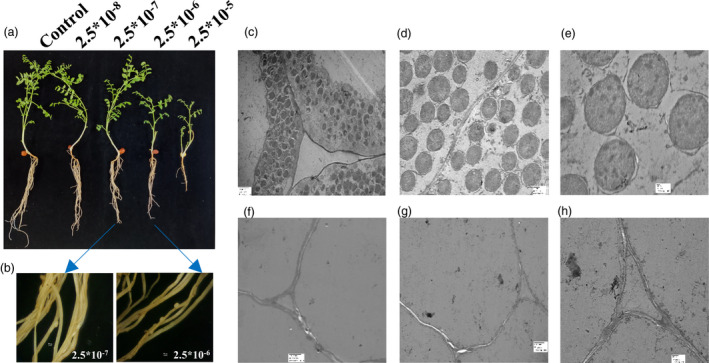
Effect of exogenous cytokinin treatment in chickpea seedlings (a) Phenotypic appearance of 30‐day‐old chickpea seedlings under different concentration of 6‐BA [2.5 × 10^−8^ to 2.5 × 10^−5M^]. (b) Stereo zoom microscopic images are showing pseudo‐nodules observed in 2.5 × 10^−7M^ and 2.5 × 10^−6M^ (scale bar = 50 pixel) without rhizobia infection. Control: no treatment with cytokinin and rhizobia; 6‐BA concentration: 2.5 × 10^−8^ to 2.5 × 10^−5M^. (c‐e) TEM images of true chickpea containing bacteriods with *M. ciceri* infection. [Scale bar = 2 µm, 500nm and 100 nm, respectively, and magnification = 1000×, 2500× and 6000×, respectively]. (F‐H) TEM images of chickpea pseudo‐nodules in cytokinin concentration of 2.5 × 10^−7M^ in the absence of *M. ciceri* infection. [Scale bar = 2 µm 2 µm and 500nm, respectively and magnification = 1000×, 1000× and 2500×, respectively]. Experiments were carried in three biological replicates in ten chickpea seedlings.

Four‐day‐old chickpea seedlings treated exogenously with varying concentration of cytokinin resulted in a significant change in root phenotype. Treatment with high concentration of cytokinin, that is 2.5 × 10^−5 M^ resulted in stunted root, whereas at low concentration (2.5 × 10^−8 M^), no marked difference from control was observed (Figure 1a). Interestingly, at 2.5 × 10^−6^ to 2.5 × 10^−7^ M concentrations, nodule‐like structures (pseudo‐nodules) were observed even in the absence of rhizobia (Figure 1b), which indicated that cytokinin was sufficient to induce nodule organogenesis. The true nitrogen‐fixing nodules showed bacterial colonization (Figure 1c–e), whereas pseudo‐nodules were devoid of rhizobia as represented by transmission electron microscopy (TEM) images (Figure 1f–h). This observation was supported by evidences of pseudo‐nodule formation upon exogenous cytokinin treatment in other legumes (Gauthier‐Coles *et al*., 2019). To further decipher the role of cytokinin at the molecular level, its downstream signalling components, the two‐component system (TCS) genes, were identified, from chickpea and related legumes at genome‐wide level.

### Global identification and annotation of TCS genes in legumes

Genome‐wide identification of the TCS genes from chickpea was carried out using the available whole‐genome sequences of kabuli and desi chickpea types. After removing redundancy, a set of 67 TCS genes were identified in chickpea that were categorized into (Figure S1, Table S1) CaHKs, CaHPs and CaRRs. Nineteen *Ca*HK(L) proteins were found that were further divided into two groups: *Ca*HKs containing conserved histidine kinase domains and *Ca*HKLs possessing diverged histidine kinase‐like domains (Table S1–S2). Eight HPs were identified of which 6HPs had conserved HPt domains and 2PHPs had pseudo‐HPt domains in which His phosphorylation site was absent (Table S1–S2). Additionally, 40 CaRR proteins were identified that were classified into four categories, viz: 10 TypeA RRs, 16 TypeB RRs, 9 TypeC RRs and 5 Pseudo RRs based on the composition of their conserved domains and motifs (Table S1–S2). Further, to obtain a holistic overview of the TCS family in legumes, genome‐wide identification of TCS genes was done from *M. truncatula* and *C. cajan* genomes that led to identification of 96 and 75 TCS members, respectively (Figure S1, Table S2). *Medicago* had 21 MtHKs and six MtHPs and two MtPHPs. Also, there were 67 RRs classified as 11 TypeA RRs, 35 TypeB RRs, 12 TypeC RRs and 9 Pseudo RRs. *C. cajan* was found to have 75 TCS members containing 20 CcHKs, 8HPs, 2PHPs and 45 CcRRs of which 12 were TypeA RRs, 16 TypeB RRs, 11 TypeC RRs and 6 Pseudo RRs. Interestingly, the HKs were found to be conserved among the 3 legumes (Table S2). Amino acid sequences of TCS members of the 3 legumes were aligned using MUSCLE. Pfam/CDD were used to annotate the signature functional domains. Protein structure, domain and motif alignment analysis revealed that the kinase domain of HKs, HPt in phosphotransferases, Rec and Myb in TypeB RRs and rec in other RRs were very well conserved. Representative members from each class of TCS were depicted to highlight the important functional domains (Figure S2). Through these structural analyses, it was clear that members of a particular class followed a similar pattern of organization at proteomic levels across legumes.

### Phylogenetic analysis of TCS members from chickpea, *Medicago*, *C. cajan*, soybean, Arabidopsis and rice

Phylogenetic analysis based on amino acid sequences was carried out separately for each class of TCS members. Phylogenetic relationships between the HK(L) of chickpea, *Arabidopsis*, rice, *Medicago* and *C. cajan* reveal presence of 7 subclades which were based on the presence of specific domains (Figure S3). The HKs phylogram showed two major subclades: the cytokinin receptor subclade containing 4 CaHKs (*CaHK7, 14, 18* and *19*) and the ethylene receptor subclade comprising of 5 members from chickpea (*CaHK2, 5, 6, 12* and *13)*. The *AHK4* also known as *CRE1* in Arabidopsis and *Medicago* was found in the cytokinin receptor family, and *CaHK19, MtHK21* and *MtHK11* were present in the same subclade. Other three cytokinin receptor CaHKs (*CaHK7, 14* and *18*) were present in a separate subclade clustering along with *AHK2* and *AHK3*. Phylogenetic relationships among the HPs of the above 5 species revealed that HPs were grouped into subclades with PHPs forming a distinct subclade (Figure S4). An interesting observation on phylogram of HPs was that all the members of *Oryza sativa* fall in a distinct subclade. Cytokinin signalling positive regulator, *AHP1*, showed a closer relationship with *CaHP1*, *MtHP3* and *CcHP5‐6*, while *AHP2, 3* and *5* had a closer relationship with *CaHP5*, *MtHP2* and *CcHP2,* respectively. Similarly, cytokinin signalling negative regulator *AHP4* showed a closer relationship with *CaHP4*, *CaHP6*, *MtHP4‐5* and *CcHP3‐4*. Furthermore, phylogenetic analysis of the 268 RR proteins classified them into 10 clades (Figure S5). Diversity in response regulators was markedly noticeable through its phylogenetic tree. TypeB RRs that generally comprise of TFs were seen to be quite complex and were further classified into six subgroups (I‐VI) where the maximum number of RRs were present in the TypeB‐I RR subclade. Overall, most of the subclades had representative members from legumes as well as Arabidopsis and rice. Interestingly, species‐specific and legume‐specific clades were also observed (TypeB‐III, IV, V and VI RRs). This clearly indicated the conservation among TCS members of legumes and divergence from monocotyledonous rice.

### Gene duplication and evolutionary analysis of TCS members between legumes and non‐legumes

The Plant Genome Duplication Database was used to deduce the duplicated pairs of TCS members (paralogs) within chickpea, *Medicago* and *C. cajan* which revealed 15, 22 and 6 pairs, respectively (Table S3A). Further, orthologous pairs between chickpea–*Medicago* (75 pairs), chickpea–*C. cajan* (24) and chickpea–soybean (172) were also identified (Table S3B). Comparison of *Medicago* with *C.cajan* and soybean identified 18 and 144 orthologous pairs, respectively (Table S3C). Interestingly, a greater number of orthologs were present in chickpea*–C. cajan* (24) in comparison to *Medicago*–*C. cajan* (18). Comparison of orthologous pairs between chickpea and *Medicago* showed presence of maximum orthologous pairs on chromosomes Ca4‐Mt1 (12 pairs) which corroborates the previously published syntenic relationship between chickpea and *Medicago* genome (Figure S6, Table S4). Presence of orthologs in large numbers and synteny analysis depicted a close relationship of chickpea with *Medicago* and soybean and a common ancestry within the legumes. In‐depth analysis of the pattern of evolution and divergence of TCS within non‐legume species was also performed. The number of TCS ortholog pairs between Arabidopsis–chickpea, Arabidopsis–*Medicago* and Arabidopsis–*C. cajan* were 20, 26 and 13, respectively (Table S5). Relative Ks values of TCS pairs (obtained from the PGDD) were used to calculate the divergence time within legumes and Arabidopsis as described in materials and methods. The graph peaks at Ks values of ˜2.0–2.1 which corresponded to ˜133 to 140 Mya represented the time of divergence of the 3 legumes from Arabidopsis. Similarly, the relative Ks values of ˜0.3–0.4 (Figure S6d–e), ˜0.5–0.6 (Figure S6d) and ˜0.7–0.8 (Figure S6d and f), for orthologs between chickpea–*Medicago,* chickpea–soybean and chickpea–*C. cajan* corresponded to the divergence time of ˜25–33, ˜42–50 and ˜58–66.6 Mya of chickpea from *Medicago*, soybean and *C.cajan,* respectively (Figure S6d–f). These data are in accordance with previous findings that chickpea diverged most recently from *Medicago,* followed by its divergence from soybean, *C. cajan* and Arabidopsis.

The distribution of the Ka/Ks ratios of HKs revealed median values ranging between 0.09 and 0.18. The median of Ka/Ks ratios for RRs ranged from 0.23 to 0.37 (Figure S7a,b). The lower value of Ka/Ks for HKs indicated that strong purifying selection acted during evolution while the higher Ka/Ks ratio of RRs indicated more divergence in comparison to HKs (Figure S7c,d). This was further validated by analysing the ratio of the mean Ka values (RR/HK) of the orthologous pairs which were found to be ˜2, whereas the mean Ks values were ˜1 (*P*‐value‐0.00001) (Table S3). The higher mean Ka value of RRs along with lower mean Ks value denoted the higher levels of divergence within RRs in comparison to HKs (Table S3).

Analysis of orthologs between Ca_At, Ca_Gm and Ca_Mt revealed that out of 19 chickpea HKs, 14 were common with Arabidopsis, 17 with soybean and 18 with *Medicago*. Overall, the Ka/Ks values for each of these pairs were fairly low (<0.23) indicating that the HK family was undergoing purifying selection and was quite conserved (lower Ka/Ks value) (Figure S7c, Table S3 and Table S5). On the other hand, analysis of RRs revealed that between chickpea and soybean there were 24 orthologs and 25 between chickpea and *Medicago*. However, with Arabidopsis only 4 out of 40 RRs (10%) formed orthologous pairs, thereby clearly indicating a legume‐specific duplication and diversification of RRs (Figure S7d).

### Expression analysis of TCS members in various tissues of chickpea, *Medicago* and *C. cajan*

*In silico* tissue‐specific expression analysis of TCS members from chickpea, *Medicago* and *C. cajan* was carried out using the RNA‐seq data. This revealed a differential transcriptional expression pattern of the TCS members in all 3 species. The members of cytokinin receptors and ethylene receptors showed high expression, whereas the members of cytokinin insensitive families (AHK1, CKI1 and CKI2) were found to be lowly expressing in all three legumes. Further, few members of TypeA and TypeB RRs also showed enhanced expression. Additionally, expression of 6HPs in chickpea, *Medicago* and *C. cajan* was found to be highly reduced. Similarly, TypeB‐II RRs and TypeC RRs were also down‐regulated in comparison to other RRs, while members belonging to TypeA, TypeB‐I RR and TypeB‐VI RR clades showed intermediate expression (Figure S8a–c). TypeB PRRs showed fairly high expression except in root and nodule tissues, and Pseudo RR’s showed high expression across all the tissues. CaRR2, CaRR13 and *Ca*RR18 showed fairly high expression across all the tissues.

To validate the *in silico* expression values, 18 highly expressing, CaTCS genes (out of 67) were selected, and their real‐time expression analysis was carried out in various tissues of chickpea (flower, leaf, nodule, root, seed and seedling). Most genes showed high expression in leaves and low expression in seed tissue (Figure S8d). Since TCS genes play a crucial role in cytokinin‐mediated nodule formation, the expression of the 18 TCS members was validated in nodule tissues at various stages of development. It was observed that TCS genes such as *CaHP1, CaHP3, CaPRR5, CaRR3* and *CaRR18* were found to have significantly high expression throughout nodulation (Figure S8e). The expression of TCS genes was induced onwards at 24 hpi and steadily maintained up to 3 weeks. However, expression of RRs was higher as compared to HKs in mature nodules. Overall, it indicated that *Ca*TCS genes played diverse roles in all tissues including nodules.

The expression analysis data were further used to analyse the orthologous/paralogous gene pairs in order to deduce evolutionary significance based on structural and functional relevance. The paralogous pairs of cytokinin receptors were also orthologous pairs between chickpea and *Medicago* (*CaHK7‐CaHK18* and *MtHK20‐MtHK12*, *CaHK7‐MtHK20* and *CaHK18‐MtHK12*) implicating their importance in cytokinin signalling (Table S3A‐C). Similar tissue‐specific expression patterns were observed in orthologous pairs (*Ca*HK7‐*Mt*HK20 and *CaHK18‐MtHK12*) while paralogs (*CaHK7‐CaHK18* and *MtHK12‐MtHK20*) showed antagonistic expression depicting subfunctionalization. However, considering another set of paralogous pairs/orthologous pairs of cytokinin insensitive family (CKI) (*CaHK4‐CaHK8*, *MtHK1‐MtHK13 /* *CaHK8‐MtHK1* and *CaHK4‐MtHK13*), the expression patterns of orthologous and paralogous pairs were similar depicting no functional diversification in members of the cytokinin insensitive family (Figure S8a–c, Table S3A–C). Additionally, ethylene receptor subfamily (I and II) and phytochrome subfamily were also explored in three legumes. The paralogs of an ethylene receptor family were also orthologs among chickpea, *Medicago* and *C. cajan* (Figure S8a–c and Table S3A–C).

The paralog pairs of TypeA RR in chickpea (*CaRR12‐CaRR3*) were also orthologous in *Medicago* (*MtRR14‐MtRR32*) and *C. cajan* (*CcRR6* and *CcRR37*). Again, a similar tissue‐specific pattern was observed in orthologs (*CaRR12‐MtRR32‐CcRR37*) with a high expression in nodule and low in seed and pod, whereas paralog (*CaRR3*) showed high expression in few seed stages and low expression in nodule. TypeB RRs, *CaRR13* and *CaRR18* were paralogous to *CaRR15* and *CaRR14,* respectively. They were also orthologous to *MtRR21‐MtRR2* and *MtRR34‐MtRR7*. The orthologous pairs showed similar expression whereas paralogs *CaRR13‐CaRR15* and *CaRR18‐CaRR14* showed antagonistic expression (Figure S8a–c and Table S3A–C). This was true in most of the duplicated TCS pairs, but not in *Medicago* as the major evolution of members was through local gene duplications.

Overall, the above analysis based on phylogenetic, evolutionary and expression studies indicated that HKs and RRs played important roles in nodulation. Since HKs were found to be highly conserved and their role in nodulation is well established in model legumes such as *L. japonicus* and *M. sativa* (Gonzalez‐Rizzo *et al*., 2006; Murray *et al*., 2007; Tirichine *et al*., 2007), their functional characterization to validate their receptor activity was carried out in chickpea. Contrastingly, RRs were found to be more diverse and limited studies are available investigating their role in nodulation; therefore, detailed functional characterization of a chickpea TypeB RR member was carried out.

### Characterization of chickpea cytokinin receptors (CaHKs) involved in root nodulation

To establish the role of cytokinin receptors CaHKs (*CaHK7, CaHK14, CaHK18* and *CaHK19*), in cytokinin signalling and nodule organogenesis, their expression profiles were analysed in chickpea seedlings treated with 2.5 × 10^−6^ and 2.5 × 10^−7^ M cytokinin. Additionally, the expression of *CaNIN* (NODULE INCEPTION) was also analysed since NIN is a nodule‐specific TF that has been reported in several previous investigations (Gonzalez‐Rizzo *et al*., 2006; Heckmann *et al*., 2011; Plet *et al*., 2011) to have elevated expressions after exogenous application of cytokinin and also lead to formation of nodule‐like structures (pseudo‐nodules).

**Figure 2:**
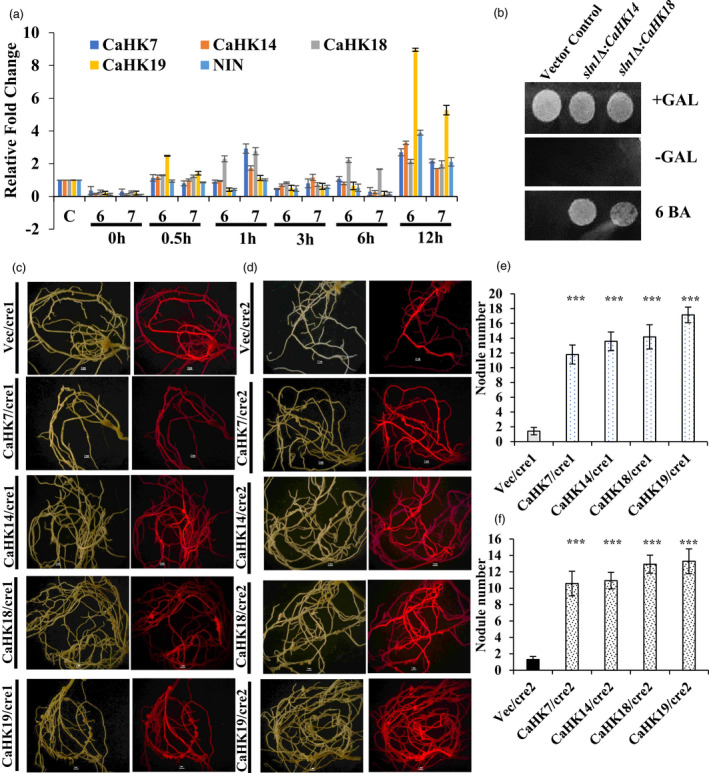
qRT‐PCR expression and heterologous expression of *CaHK*s. (a) Bar diagram represents the expression profiles of *CaHK*s and *CaNIN* after treatment of chickpea seedlings with 6‐BA at two concentrations (6 denotes 2.5 × 10^−6^ M and 7 denotes 2.5 × 10^‐7^ M) at 0 min, 30 min, 1h, 3h, 6h and 12h. ‘C’ denotes untreated control plants. The experiments were carried out in triplicates (*n *= 15 plants) and expressed as means ± S.E. (b) Yeast drop assay for complementation of yeast mutant *sln1∆* with *CaHK14* and *CaHK18*. (c) Complementation of *∆cre1‐1 Medicago* mutants with *CaHK*s. (d) Complementation of *∆cre1‐2 Medicago* mutants with *CaHK*s. Panel 1 represents visualization in white light, and panel 2 represents visualization under red fluorescence. (e) Bar diagram represents the number of nodules in the *CaHKs* complemented *∆cre1‐1* mutant lines (*** represents t‐test *P*‐value ≤ 0.0001). (f) Bar diagram represents the number of nodules in the *CaHKs* complemented *∆cre1‐2* mutant lines (*** represents t‐test *P*‐value ≤ 0.0001). The experiments were carried in triplicates (*n *= 15 plants) and expressed as means ± S.E.

At both concentrations of cytokinin, the expression of *CaHK*s started increasing 12 h post‐treatment; however, the level of elevation was far greater upon treatment with higher concentration of cytokinin (2.5 × 10^−6 M^) (Figure 2a). Among the CaHKs, *CaHK19* showed maximum induction in response to cytokinin treatment. Interestingly, the expression of *CaNIN* was also found to be similarly elevated as *CaHK*s at 12 h post‐treatment (Figure 2a). The co‐expression pattern of *CaHK*s and *CaNIN* indicated a link between cytokinin perception and downstream *CaNIN* expression.

Further, to functionally validate *CaHKs* as true cytokinin receptors, *CaHK* genes were used to complement the yeast mutant *sln1Δ*. The *Sln1* gene is an osmosensor histidine kinase which regulates the changes in turgor pressure (Reiser *et al*., 2003). The yeast mutant lines were complemented by all the 4 CaHKs; however, only *CaHK14* and *CaHK18* (*Δsln1: CaHK14* and *Δsln1: CaHK18*) revived efficiently in cytokinin‐dependent manner as compared to mutant and vector control (Figure 2b). Further, to establish the cytokinin receptor activity of the CaHKs in nodules, complementation of the nodulation‐deficient *Medicago* mutant, *cre1* was carried out. The *cre1* mutant has two lines *cre1‐1* and *cre1‐2*. *cre1‐1* has 3 single nucleotide mutations leading to the introduction of a stop codon in the kinase domain and *cre1‐2* has mutation in the conserved residue of G2 motif (Plet *et al*., 2011). Upon complementation of the *cre1* mutant lines by all four cytokinin receptors (CaHKs) through hairy root transformation, the nodule‐deficient phenotype of *cre1‐1* and *cre1‐2* mutants was restored (Figure 2c–d). A significantly high number of nodules were observed in all the CaHKs complemented lines (˜11–18 nodules) in comparison to the vector control (˜1–2 nodules) with the highest increase obtained with *CaHK19* (˜18 nodules) (Figure 2e–f).

### Functional characterization of *CaRR13*: Expression analysis of the chickpea TypeB RRs

**Figure 3:**
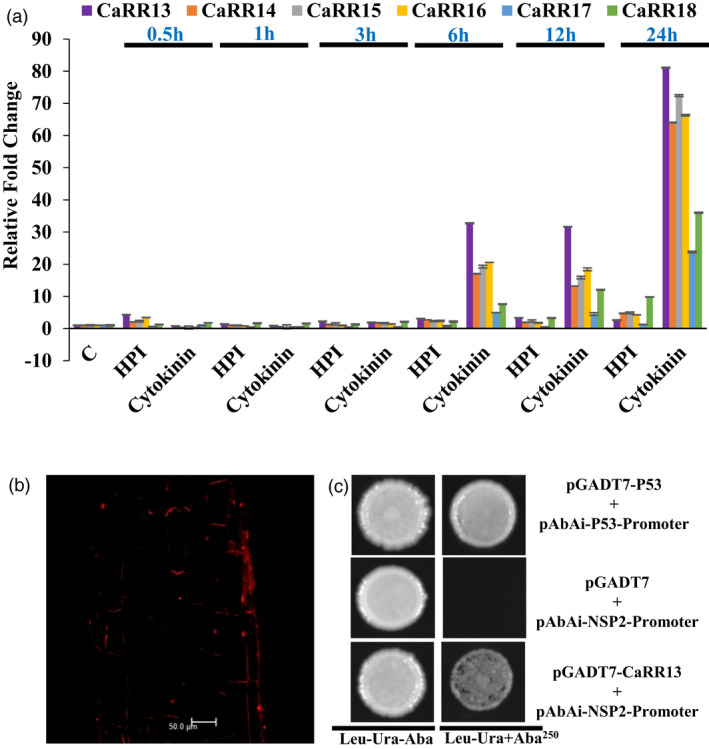
Functional characterization of *CaRR13*. (a) Bar diagram represents the expression profiles of *CaRR*s during control (c), *M. ciceri* treatment (expressed as HPI) and cytokinin (2.5 × 10^−7M^ 6‐BA). The experiments were carried out in triplicates and expressed as means ± S.E. (b) Subcellular localization of *CaRR13* (c) Interaction of *CaRR13* with *NSP2* promoter using yeast one hybrid analysis. Strong interaction was observed on Ura and Leu deficient plates in the presence of aureobasidin A (AbA250). P53 promoter and AD‐Rec‐P53 were used as a positive control.

Recent studies (Tan *et al*., 2020) have indicated that TypeB RRs may be critically involved in coordinating the expression of early nodulation genes. Moreover, the evolutionary analysis also indicated that RR components of TCS family were undergoing structural and functional diversification. Within RRs, the TypeB RRs were the most variable thereby making them interesting candidates for functional characterization. An earlier study in *Medicago* (Ariel *et al*., 2012) had reported that *MtRR1* (*MtRR20* in this study) interacts with the promoter of *NSP2* and acts as a TF. Hence, the chickpea TypeB RRs falling in the same clade as *MtRR20* were selected and the qRT‐PCR‐based expression analysis of *CaRR13*, *CaRR14*, *CaRR15*, *CaRR16*, *CaRR17* and *CaRR18* was done after 2.5x10^−6 M^ cytokinin treatment and *M*. *ciceri* infection. The analysis revealed that all the TypeB RRs were low expressing during early stages of infection; however, their expression started elevating upon cytokinin treatment at 6h and continued to increase up to 24 hpt (Figure 3a). Among the TypeB RRs, *CaRR13* showed maximum induction of expression and was selected as the potential candidate for further characterization. *CaRR13* was found to be a 616‐amino acid long protein present in a legume‐specific clade with *Medicago* *(MtRR1, 2, 3, 20, 21* and *53), C. cajan (CcRR27* and *30)* and soybean (*GmRR26, 27, 28* and *31*) (Figure S9a–d). Additionally, it had a paralog, *CaRR15* in the same clade (Figure S9b). Domain analysis revealed that it possesses a REC domain, Myb domain and a nuclear localization signal (Figure S9c). Further, it showed high per cent identity with the MtRR20 and MtRR21 (Figure S9d).

### Functional characterization of *CaRR13*: Subcellular localization and Y1H analysis indicates *CaRR13* to be a TF

In order to examine the subcellular localization of *CaRR13,* the full‐length CDS of *CaRR13* was cloned into pUBI‐dsRed vector and transformed into chickpea roots using hairy root transformation. Transformed chickpea roots were harvested and checked under confocal microscopy, which revealed subcellular localization of *CaRR13* in the nucleus (Figure 3b). Further, the yeast one hybrid (Y1H) assay was conducted to check the interaction between the *NSP2* promoter and *CaRR13*. The yeast TFs can influence the promoter sequence recognition and may give false results; therefore, minimum inhibitory concentration of AbA was measured. The minimal inhibitory concentration of AbA was identified to be 250ng/ml (Figure 3c). The Y1H gold strain containing pBait‐AbAi was transformed using the prey, pGADT7‐*CaRR13,* and checked for interaction on SD/‐Leu/−Ura/+AbA^250^ media plates. Y1H analysis clearly showed interaction of *CaRR13* with the *NSP2* promoter. These results indicated the function of the *CaRR13* as a TF functioning with the help of NSP2 as a putative coupler between cytokinin signalling and nodulation.

### Functional characterization of *CaRR13*: Role in nodule organogenesis

**Figure 4:**
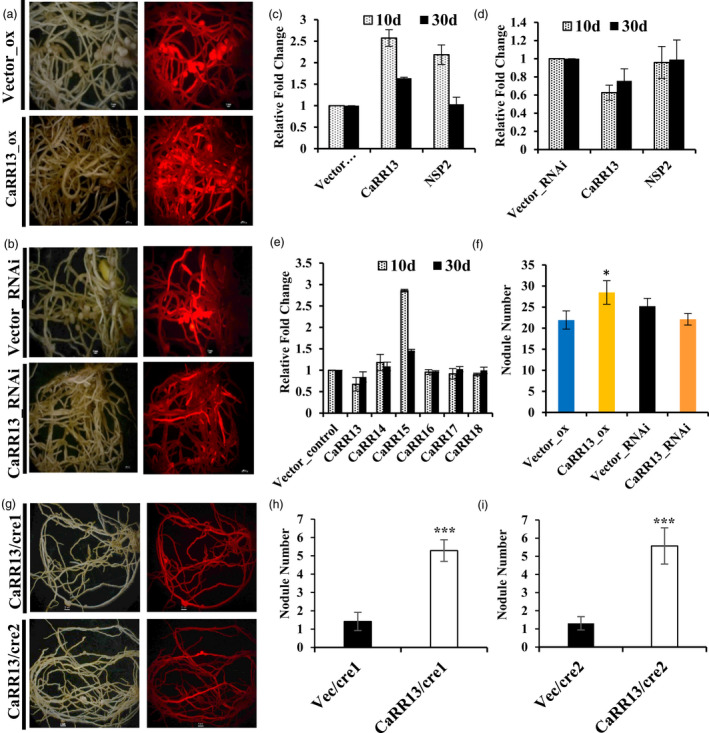
Functional depiction of role of *CaRR13* during root nodulation in chickpea. (a) Overexpression of *CaRR13* in chickpea through hairy root transformation. (b) Knockdown analysis using RNAi of *CaRR13* in chickpea through hairy root transformation. (c) Relative quantification of *CaRR13 and NSP2* expression in overexpression lines compared to respective vector controls. (d) Relative quantification of *CaRR13 and NSP2* expression in RNAi lines compared to respective vector controls. (e) The expression profiling of TypeB RRs in *CaRR13* RNAi knockdown lines. (f) Change in nodule number upon overexpression and RNAi lines of *CaRR13* in comparison to respective vector control (* represents t‐test *P*‐value ≤ 0.05). (g) Complementation of *∆cre1‐1* and *∆cre1‐2 Medicago* mutants with *CaRR13* (h) Bar diagram represents the number of nodules in the *CaRR13* complemented *∆cre1‐1* mutant lines (*** represents t‐test *P*‐value ≤ 0.0001). (i) Bar diagram represents the number of nodules in the *CaRR13* complemented *∆cre1‐2* mutant lines (*** represents t‐test *P*‐value ≤ 0.0001). The experiments were carried out in triplicates (*n *= 15 plants) and expressed as means ± SE.

For further investigating the role of *CaRR13* in nodulation, its overexpression lines were generated via the hairy root transformation (Figure 4a–f). The nodule numbers in the plants overexpressing *CaRR13* increased up to ˜1.3 times of the control plants (*P *< 0.05 t‐test) (Figure 4f). Further, the expression levels of CaRR13 in overexpression lines was higher at 10 day (˜2.6 times) and at 30 day (˜1.6 times) as compared to the respective vector control (Figure 4c). The expression level of an important nodule signalling TF, NSP2 also known to interact with *CaRR13,* was also found to be elevated in the overexpression lines at 10 day (˜2.1 times), but decreased to levels comparable with control at the 30‐day nodule stage (˜1.0 times) (Figure 4c).

Further, the knockdown (RNAi) lines of *CaRR13* were generated via hairy root transformation. Analysis of these lines revealed a decrease in expression of the *CaRR13* gene at 10 day (˜.62 times) and at 30 day (˜.75 times) compared to control (Figure 4d). Additionally, expression of the *NSP2* gene remained constant in the RNAi lines (0.95–0.99 times). The decrease in expression of *CaRR13* resulted in a decrease in the nodule number of knockdown lines to 0.88 times of vector control (Figure 4f). The knockdown lines of CaRR13 did not result in a significant decrease in nodule number indicating a partial redundancy in the function of RRs governing root nodulation. To check the partial functional redundancy, a qRT‐PCR expression analysis of the related TypeB RRs was performed using the generated RNAi knockdown lines. This revealed that *CaRR14* (to some extent) and mainly *CaRR15* redundantly compensated the function of *CaRR13* in RNAi lines, whereas the expression of other RRs (*CaRR16, 17, 18*) remained unchanged (Figure 4d). This clearly indicated a very tight regulation of nodule number in chickpea.

To further establish the role of *CaRR13* in the cytokinin response pathway, complementation of the *Mtcre1* mutants was done with *CaRR1*3. *CaRR13* was transformed into the *cre1‐1* and *cre1‐2* mutant lines by hairy root transformation to check its effect on the nodulation‐deficient phenotype of the mutants (Figure 4g–i). The mutant lines expressing the *CaRR13* resulted in the restoration of the nodule‐deficient phenotype due to *CaRR13* expression in the mutant background. A significant difference was observed in the resulting phenotype, that is nodule number after complementation with *CaHKs* and *CaRR13* (Figure 2c–f and Figure 4g–i). The number of nodules formed in the *CaRR13* complemented *cre1‐1*(˜5.2) and *cre1‐2* (˜5.5) was intermediate between vector control (˜1.4 and 1.3) and *CaHK*s complemented lines (˜14.1 and 11.9) (Figure 2c–f and Figure 4g–i).

## Discussion

Symbiotic interactions of rhizobia with legumes involve perception of flavonoid‐like substances from root exudates by bacteria and, in turn, bacteria releases Nod factors. Release of Nod factors induces an array of phytohormones such as auxin and cytokinin in roots, which play a key role in root nodule formation. The role of cytokinin in nodule organogenesis is fairly well elucidated. During nodulation, cytokinin responsive genes have been reported to be up‐regulated (Chen *et al*., 2012; Liu *et al*., 2019; Mortier *et al*., 2014). In our study, pseudo‐nodulation was observed after exogenous cytokinin treatment of 2.5 × 10^−6^ and 2.5 × 10^−7^ 6‐BA to the crop plant chickpea. Similar observation of pseudo‐nodulation had been reported earlier in *Lotus japonicus* (Heckmann *et al*., 2011) and several other legumes (Gauthier‐Coles *et al*., 2019). Cytokinin acts downstream of Nod factor signalling and induces the genes required for cortical cell activation and division, independent of bacterial infection and colonization (Heckmann *et al*., 2011). Cytokinin signalling is manifested through the TCS in plants. Previous investigations have shown that members of the TCS sensor class (HKs) were significantly active during the cortical cell division, which lead to nodule organogenesis (Held *et al*., 2014). On the other hand, the role of important class of RRs is also being explored in model legumes (Ariel *et al*., 2012; Gonzalez‐Rizzo *et al*., 2006; Op den Camp *et al*., 2011; Tan *et al*., 2020). Therefore, in this study, global identification of all classes of TCS was carried out in the important crop legume, that is, chickpea. Moreover, to facilitate the comparative analysis, a genome‐wide identification of the TCS members was also done from the model legume *Medicago* and crop legume pigeonpea (*C. cajan*) where this information was not available. Among the 3 legumes, the maximum number of TCS members were identified in *Medicago* primarily due to the massive local gene duplications reported in *Medicago* (Young *et al*., 2011). The number of TCS members in each of these species was directly correlated with the total number of genes in the respective species (Table S6). Our systematic analysis of TCS genes in the 3 species with respect to the number of classes, presence of domains and motifs were in accordance with those reported in previous studies. The functional annotation as well as the *in silico* transcriptome analysis provided insights into the involvement of TCS genes in diverse plant functions.

Phylogeny based on HKs showed classification into subclades which could be distinguished based on the presence of similar functional domains. Phylogenetic analysis of HKs clearly distinguished the clade of CHASE domain containing cytokinin receptors. This clade contained the well‐characterized member (AHK4, CRE1) which has been shown to be the master receptor of cytokinin signalling (Boivin *et al*., 2016; Gonzalez‐Rizzo *et al*., 2006). The chickpea ortholog *CaHK19* was in the close proximity to AHK4 which was found to be the master spigot of cytokinin perception in chickpea. Phylogeny of RRs indicated distinct clades some of which had members only from one species, for example the Arabidopsis specific clade (TypeB‐III RRs), rice clade (TypeB‐IV and V RRs) and legume‐specific clade (TypeB‐VI RRs). Interestingly, a TypeB RR from *Medicago, MtRR1 (MtRR20* in present study*)* acts as TF and binds to the promoter of *NSP2* gene also forms a distinct legume‐specific subclade (Ariel *et al*., 2012; Gonzalez‐Rizzo *et al*., 2006).

The Ks value peaks of orthologous pairs between legumes and *Arabidopsis* was ˜2.0–2.1 revealing the occurrence of duplication ˜133–140 Mya. The peaks probably depicted the ‘gamma triplication or whole genome hexaploidy’ commonly shared by all core eudicots (Jain *et al*., 2013; Varshney *et al*., 2011; Young *et al*., 2011). The major peaks of TCS orthologs at ˜0.7 among legumes corresponded to a common duplication event (˜47 Mya) during the origin of papilionoid family. This duplication event coincides with the Cretaceous–Paleogene extinction event which is a hallmark of global extinction event (Figure S6). The comprehensive analysis of orthologous pairs as well as the evolutionary analysis of HKs and RRs revealed that 14 out of 19 HKs were conserved between chickpea and Arabidopsis, whereas in case of RRs, only 4 out of 40 RRs were found to be conserved. Moreover, even within legumes, the RRs were found to be more diverged (25 out of 40 between chickpea and *Medicago*). This analysis indicates diversification of RRs as compared to HKs. Additionally, genome‐wide median Ka/Ks values and higher Ka values of RRs as compared to HKs depicted a greater number of non‐synonymous substitutions in RRs compared to HKs resulting in amino acid changes and hence an increased probability of neofunctionalization. Therefore, it can be inferred that cytokinin receptors remain conserved, but RRs evolved to perform diverse functions. Additionally, 2–3 pair of RRs between chickpea and soybean showed Ka/Ks value higher than 1 suggesting the recent positive selection. This might be due to recent WGD in soybean ˜13 Mya (Table S3). Through phylogeny and evolutionary analysis, it became evident that TypeB RRs diverged between monocot and dicot and further among legumes depicting the complexity and the diverse functional roles of TypeB RRs in TCS signalling (Figure S5 and Figure S7). Therefore, in this study, TypeB RRs were explored to understand their response to cytokinin and their role during root nodulation.

Though extensive studies of TCS are present in model legumes, no such studies have been performed in crop legumes such as chickpea. Therefore, in our study, we explored the role of key cytokinin receptors (CaHKs) as well as CaRRs. Through whole‐genome analysis, four cytokinin responsive CaHKs (CaHK7, 14, 18 and 19) were identified (Ariel *et al*., 2012; Boivin *et al*., 2016; Gonzalez‐Rizzo *et al*., 2006; Held *et al*., 2014). Expression analysis showed the up‐regulation of CaHKs specifically *CaHK19*, an ortholog of *MtCRE1 and LHK1,* after 6h of exogenous cytokinin treatment and *M. ciceri* infection. Additionally, *CaNIN* was also found to be up‐regulated in a similar manner. NIN is a nodule‐specific TF that is involved in nodule organogenesis. It has been shown in earlier studies (Gonzalez‐Rizzo *et al*., 2006; Heckmann *et al*., 2011; Plet *et al*., 2011) that exogenous application of cytokinin can result in elevated NIN expression and also formation of nodule‐like structures (pseudo‐nodules). Further, it is also well established that the induction of NIN expression by cytokinin requires the cytokinin receptor 1 (*CRE1*), for nodule organogenesis (Gonzalez‐Rizzo *et al*., 2006; Plet *et al*., 2011; van Zeijl et al., 2015). A similar result was obtained in our study of chickpea where induction of expression of CaHKs and *CaNIN* exhibited a similar trend after treatment with cytokinin. The co‐expression pattern of CaHKs and *CaNIN* clearly indicated a link between cytokinin perception and the downstream expression of *CaNIN* in chickpea as earlier established in *Medicago*. Further, molecular evidence in support of the above was provided by a recent study performed by Liu *et al*., 2019 which showed that a remote upstream regulatory sequence (the CE region), located 5‐kb upstream of the NIN start codon, contained several putative cytokinin response elements and was essential for nodule primordia formation (Liu *et al*., 2019). This provided strong evidence to support that cytokinin signalling triggered *NIN* expression to initiate primordium formation.

Expression analysis of CaHKs also revealed highest expression of *CaHK19* as compared to the other CaHKs, thereby suggesting its prominent role as a major sensor of cytokinin signalling. Earlier the role of *LHK1* as the most prominent cytokinin receptor had been established in *L. japonicus* (Murray *et al*., 2007). Another recent study showed that the *L. japonicus, LHK1* (ortholog of CaHK19) exhibited 13 to 33 times higher expression as compared to other LHKs (Held *et al*., 2014). Interestingly, *LHK1* mediates cell divisions for nodule primordium formation in a partially redundant manner with *LHK1A* and *LHK3*. Similarly, in our study of chickpea, out of the 4 identified cytokinin receptors, *CaHK19*, an ortholog of *LHK1*, was likely to be the most prominent cytokinin receptor working with the other CaHKs in a partially redundant manner. Subsequently, the four CaHKs were used for complementation of yeast mutants, but only two of the CaHKs (CaHK14 and 18) were able to successfully revive the yeast mutant strain in a cytokinin‐dependent manner. The other two CaHKs were deleterious. Similar results have been shown in *L. japonicus,* where 2 LHKs were found to be lethal for the yeast mutant strain (Held *et al*., 2014).

For further characterization of CaHKs, the *cre1‐1* and *cre1‐2* mutant lines of *Medicago* were complemented with the 4 CaHKs, which restored the nodule‐deficient phenotype of *cre1‐1* and *cre1‐2* mutants depicting that four CaHKs confers cytokinin‐induced signalling across species (Figure 2). The complemented *cre1‐1* and *cre1‐2* mutants varied with respect to number of nodules formed. Of the four CaHKs, complementation with MtCRE1 ortholog, CaHK19 showed maximum nodulation restoration phenotype. Therefore, our results have enabled functional characterization of a unique ortholog of the *Medicago* cytokinin receptor, *CaHK19,* indicated to have a prominent role in cytokinin‐mediated nodule organogenesis.

An earlier study in *Medicago* had shown *MtRR1* (ortholog of *CaRR13*), a TypeB RR, to be induced among other TypeB RRs studied during early symbiotic interactions (Gonzalez‐Rizzo *et al*., 2006). Further, another investigation established an important role of *MtRR1* as a TF that binds the cis‐elements present in the promoter region of TypeA RRs and *NSP2* gene (Ariel *et al*., 2012). To gain a better insight about the cytokinin primary response genes in chickpea nodules, we undertook the evaluation of the TypeB class of RRs in chickpea. Based on the evolutionary and *in silico* transcriptome analysis, a qRT‐PCR expression analysis of the diverse set of chickpea TypeB RRs was performed. Results showed that the chickpea TypeB RR, *CaRR13* an ortholog of *MtRR1,* showed maximum up‐regulation during cytokinin treatment and *M. ciceri* infection which was a strong indicative of the potential role of CaRR13 in nodulation. The candidate was further characterized for its TF activity by performing a Y1H analysis that confirmed its interaction with the *NSP2* promoter. Additionally, subcellular localization of CaRR13 in the nucleus indicated a strong possibility of its being a TF.

Moreover, the *in planta* functional characterization for investigating the effect of *CaRR13* on the nodule phenotype in chickpea was performed via the generation of overexpression and knockdown lines. The overexpression of *CaRR13* resulted in an increase in nodule number (˜1.3 times). The overexpression lines revealed a high level of the *NSP2* gene at an early nodule stage which normalized after 30 days depicting the *NSP2*‐driven nodule organogenesis by overexpression of *CaRR13*. In contrast to this, the knockdown lines showed a decrease in nodule number (˜.88 times) which was not very significant. The knockdown lines showed almost a similar expression of *NSP2* gene at both early and late stage with insignificant effect on nodule number. Interestingly, the qRT‐PCR analysis in knockdown lines also revealed that *CaRR15,* a paralog of *CaRR13,* to be significantly up‐regulated. This expression analysis of RNAi lines along with insignificant change in nodule number upon knockdown indicates a partial redundancy in the activity of TypeB RRs. Further, a small but significant change in nodule number upon overexpression suggested that the nodulation process was under tight regulation (Figure 4). Additionally, *CaRR13* was complemented into the *cre1* mutants of *Medicago* which showed increased nodule organogenesis although the complementation activity in terms of nodule number was intermediate between the HKs and the vector control. Earlier reports of *cre1* mutants have shown that the *cre1* mutants are able to form nodules though with reduced efficiency and delayed kinetics (Boivin *et al*., 2016). Hence, after complementation with *CaRR13,* the weak signals from the receptors could be gathered by the CaRR13 protein and resulting in downstream signalling via NSP2 to perform nodule organogenesis. Hence, in our study, the role of *CaRR13* acting as a key regulator of corticular signalling events that regulate nodule organogenesis could be established. A recent study performed in *Medicago* showed that *MtRRB3,* a TypeB RR regulates *NSP2* expression and plays a key role in cytokinin signalling during *Medicago* symbiosis (Tan *et al*., 2020). This study falls in accordance with present investigations and clearly supports findings of role of *CaRR13* in chickpea symbiosis.

This study clearly demonstrates how integrated approaches combining *in silico* tools with functional validation can be used to address important biological questions in nodulation biology. In this study, an integrated phylogenetic, evolutionary and expression analysis of TCS members in three legumes – chickpea, *Medicago* and pigeonpea led to identification of cytokinin responsive HKs and TypeB RRs. Critical role of cytokinin signalling TCS members (CaHKs and TypeB RRs) in nodulation was investigated for the first time in an important grain legume, that is chickpea. CaHKs were characterized for their receptor activity using complementation assays in yeast and *Medicago cre1* mutants. A TypeB RR, CaRR13, was shown to be a TF interacting with the promoter of an early nodulation gene, NSP2, in order to tightly regulate nodule numbers in chickpea. This comprehensive study would provide deep insights into the cytokinin‐based regulation of nodulation and serve as a foundation for designing strategies for sustainable agriculture.

## Methods

### Exogenous cytokinin treatment to chickpea seedlings

For exogenous cytokinin treatment, the ˜4‐day‐old chickpea seedlings were dipped in varying concentration of 6‐BA viz 2.5 × 10^−5^, 2.5 × 10^−6^, 2.5 × 10^−7^ and 2.5 × 10^−8 M^ and water as control. Seedlings were kept in the dark for 2 days and then potted in sterile sand supplemented with nitrogen‐free McKnight solution as the nutrient source. Plants were grown in the NIPGR plant growth facility at 22 ± 1 °C and 16‐h light/8‐h dark photoperiod. Three technical and 3 biological replicates were used for each experiment.

### Genome‐wide identification of TCS genes in chickpea, *Medicago* and pigeonpea

The available TCS protein sequences of *A. thaliana, Oryza sativa, L. japonicus, Glycine max* and *Brassica rapa* were downloaded and used as query to perform a BLAST against protein sequences of chickpea, *Medicago* and pigeonpea that were downloaded from the online databases of the respective species: kabuli chickpea (FTP server of NCBI ftp://ftp.ncbi.nlm.nih.gov/genomes/Cicer_arietinum/); desi chickpea (NIPGR Chickpea Database http://nipgr.res.in/CGAP2/download/genome_sequencing/annotation/Gene%20annotation/CGAP_v2.0/Ca_Pep_v2.0.fa); *M. truncatula* (http://www. Medicagogenome.org/downloads ); and *C. cajan* (http://gigadb.org/dataset/100028). BLASTP and HMMER were used to extract TCS sequences (at e‐value of 10) from all three legumes. In case of chickpea only non‐redundant set of TCS from kabuli and desi chickpea were retained for further analysis. All extracted sequences were analysed using SMART (http://smart.embl‐ heidelberg.de/), CDD (http://www.ncbi.nlm.nih.gov/Structure/cdd/wrpsb.cgi) and Pfam (http://pfam.janelia.org/) database for domain analysis.

### Protein structure information and phylogenetic analysis

Protein structure modelling was performed using Phyre^2^ (Kelley *et al*., 2015). For phylogenetic analysis, the multiple alignments of TCS protein sequences of chickpea, *Medicago*, *C. cajan*, soybean, Arabidopsis and rice were performed by MUSCLE program. The unrooted tree was constructed for phylogenetic analysis via neighbour‐joining (NJ) method keeping the bootstrap value 1000 in MEGA 6.0 (http://www.megasoftware.net/) and visualized through iTOL v 3 (http://itol.embl.de/).

### Digital expression analysis of TCS genes in chickpea, *Medicago* and *C. cajan*

Tissue‐specific transcriptomes of chickpea for various tissues and stresses such as leaf (SRX048833), root (SRX048832), flower bud (SRX048834), pod (SRX048835), seed (SRX125162) and nodule (SRP028391) were downloaded from SRA (Sequence Read Archive) (Garg *et al*., 2011; Kant *et al*., 2016). Reads were filtered, trimmed and mapped on the chickpea (desi) genome using TopHat2 (Kim *et al*., 2013). CuffDiff was used to determine the FPKM values which were used to visualize differential gene expression. Similarly *in silico* gene expression analysis of *Medicago* and *C. cajan* was done using LegumeIP and *C. cajan* gene expression atlas, respectively (Li *et al*., 2011; Pazhamala *et al*., 2017). Heat maps were visualized through MeV software.

### Synteny and evolutionary analysis

The estimation of segmental pairs and synteny analysis was carried out using data from Plant Genome Duplication Database (Lee *et al*., 2013). Ka/Ks values were obtained from the same. Time of divergence was calculated using the synonymous mutation rate of substitutions per synonymous site per year as per chalcone synthase gene where, T = Ks/1.5 × 10^−8^ (Koch *et al*., 2000). The evolutionary time was calculated, assuming a synonymous substitution rate per synonymous substitution of 6.1 × 10^−9^ per year (Nardon *et al*., 2005).

### Plant growth, treatments and sample harvesting for expression analysis

Chickpea seeds were surface sterilized, kept on the germination sheet for two days and then transferred to pot containing vermiculite and peat (3:1) under environmentally controlled conditions. Root, leaves, seeds and flowers were harvested and used for tissue or organ‐specific expression analysis. For nodule‐specific expression, young seedlings were infected with *M. ciceri*, transferred to the sand and supplied with nitrogen‐free McKnight’s solution.

### RNA extraction and quantitative reverse transcription PCR analysis

RNA was extracted using LiCl method (Choudhary *et al*., 2009). The first‐strand cDNA was synthesized using Bio‐Rad iScript cDNA synthesis kit following the manufacturer’s protocol. Primers were designed using Primer Express software v2.0. qRT‐PCR was performed using SYBR Green Master Mix on an ABI ViiA7 machine. EF1α and actin were used as an endogenous control. Three biological and technical replicates were kept for each experiment and 2^−ΔΔCT^ method was used to calculate the relative expression.

### Cytokinin responsive complementation of *Saccharomyces cerevisiae* mutant

All the cytokinin responsive *CaHKs* having CHASE domain were amplified and directionally cloned into yeast expression vector P415CYC (Mumberg *et al*., 1995). *AHK4* cDNA (a kind gift from Tatsuo Kakimoto Osaka University, Japan) was used as positive control. Construct containing CaHKs in vector were transformed into yeast mutant *sln1Δ* and further analysis for the response to 6‐BA was performed (Murray *et al*., 2007).

### Complementation of cre1 *Medicago* mutant lines

Full‐length cDNA sequences of *CaHK*s (*CaHK7, CaHK14, CaHK18* and *CaHK19*) and *CaRR13* were cloned into pUBIcGFP‐dsRed vector and transformed into *Agrobacterium rhizogenes* R1000 strain (Kryvoruchko *et al*., 2016). This was used for the hairy root transformation of *Mtcre1‐1* and *Mtcre1‐2* mutants (Plet *et al*., 2011). Transgenic plants were screened for red fluorescence under a stereo zoom microscope and the plants showing red fluorescence under the stereo zoom microscope were chosen for further analysis.

### Yeast 1 Hybrid (Y1H) assay

Screening for interaction of *CaRR13* with *NSP2* promoter was performed using Y1H assay. The 1.8‐kb upstream *NSP2* promoter region was PCR amplified and cloned into pAbAi vector. Then, the minimal inhibitory concentration of aureobasidin A (AbA) for the pBait (1.8‐kb *NSP2* promoter)‐AbAi‐transformed yeast strain Y1H gold was determined by growing them on the SD/−Ura/+AbA media. The full‐length CDS sequence of *CaRR13* was PCR amplified and cloned into the pGADT7 plasmid, 1 µg of construct DNA was used to transform the bait‐reporter yeast strain Y1H gold [pBait‐AbAi]. Transformed yeast was cultured on SD/‐Leu/‐URA (as a control) and SD/−Leu/‐URA/+AbA^250^ media plates.

### Hairy root transformation of chickpea

Construct carrying full‐length *CaRR13* in pUBIcGFP‐dsRed vector was used for overexpression of *CaRR13* in root and nodule of chickpea by hairy root transformation using *A. rhizogenes* R1000 strain. For generating the RNAi lines of *CaRR13*, a 200‐bp specific sequence corresponding to a region having maximum nucleotide variation from other CaRRs was amplified and cloned into pK7GWIWG2_II‐RedRoot (http://gateway.psb.ugent.be). This construct was transformed into *A. rhizogenes* R1000 strain and used for hairy root transformation in chickpea (Mandal and Sinharoy, 2018).

## Conflict of interest

The authors declare no conflict of interest.

## Author contributions

Conceived and designed the experiments: SB and MT. Performed the experiments: MT and MY. Analysed the data: MT and BS. Wrote the paper: SB and MT. MT and KN performed the computational analysis, MT and VP prepared all the figures and tables. MT, MY, BS and VP performed the chickpea and *Medicago* transformation. Critical evaluation, funding resources and all the facilities were arranged by SB.

## Supporting information


**Figure S1** Phylogenetic classification of various crop species and distribution of TCS members.
**Figure S2** Domain arrangement and structural organization.
**Figure S3** Phylogenetic analysis of HKs.
**Figure S4** Phylogenetic analysis of HPs.
**Figure S5** Phylogenetic analysis of RRs.
**Figure S6** Synteny and collinearity analysis of TCS members among legumes.
**Figure S7** Evolutionary analysis of TCS members.
**Figure S8** Digital Spatiotemporal and stress‐responsive expression analyses of TCS members and qRT‐PCR based validation.
**Figure S9** CaRR13 sequence, domain and homology.

**Table S1** List of TCS genes with their associated domain in chickpea, *Medicago* and *C. cajan*.
**Table S2** Distribution of TCS genes across various classes in the 3 legumes analysed in this study (marked by *) and other plants.
**Table S3** (A) A list of paralogs in chickpea, *Medicago* and *C. cajan* genome. (B) A list of orthologs pairs in chickpea with *Medicago*, *Cajanus* and *Glycine*. (C) A list of orthologs pairs in *Medicago* ‐*Glycine, Medicago‐Cajanus* and *Cajanus‐Glycine*.
**Table S4** Pairwise comparison of segmentally duplicated TCS pairs present on chromosome in legumes.
**Table S5** A list of orthologous pairs in legumes with Arabidopsis.
**Table S6** The ratio of total genes and TCS gene in *Medicago*‐*C. cajan*, *Medicago*‐chickpea and chickpea‐*C. cajan*.
