# Supplementary material for: Evolutionary and functional analysis of two‐component system in chickpea reveals CaRR13, a TypeB RR, as positive regulator of symbiosis
Source: Plant Biotechnol J. 2021 Sep 16;19(12):2415–27. doi: 10.1111/pbi.13649 (PMC8633487; doi:10.1111/pbi.13649)
Supplement: Supplementary file 1 — Figure S1 Phylogenetic classification of various crop species and distribution of TCS members. Figure S2 Domain arrangement and structural organization. Figure S3 Phylogenetic analysis of HKs. Figure S4 Phylogenetic analysis of HPs. Figure S5 Phylogenetic analysis of RRs. Figure S6 Synteny and collinearity analysis of TCS members among legumes. Figure S7 Evolutionary analysis of TCS members. Figure S8 Digital Spatiotemporal and stress‐responsive expression analyses of TCS members and qRT‐PCR based validation. Figure S9 CaRR13 sequence, domain and homology. [file PBI-19-2415-s001.pdf]

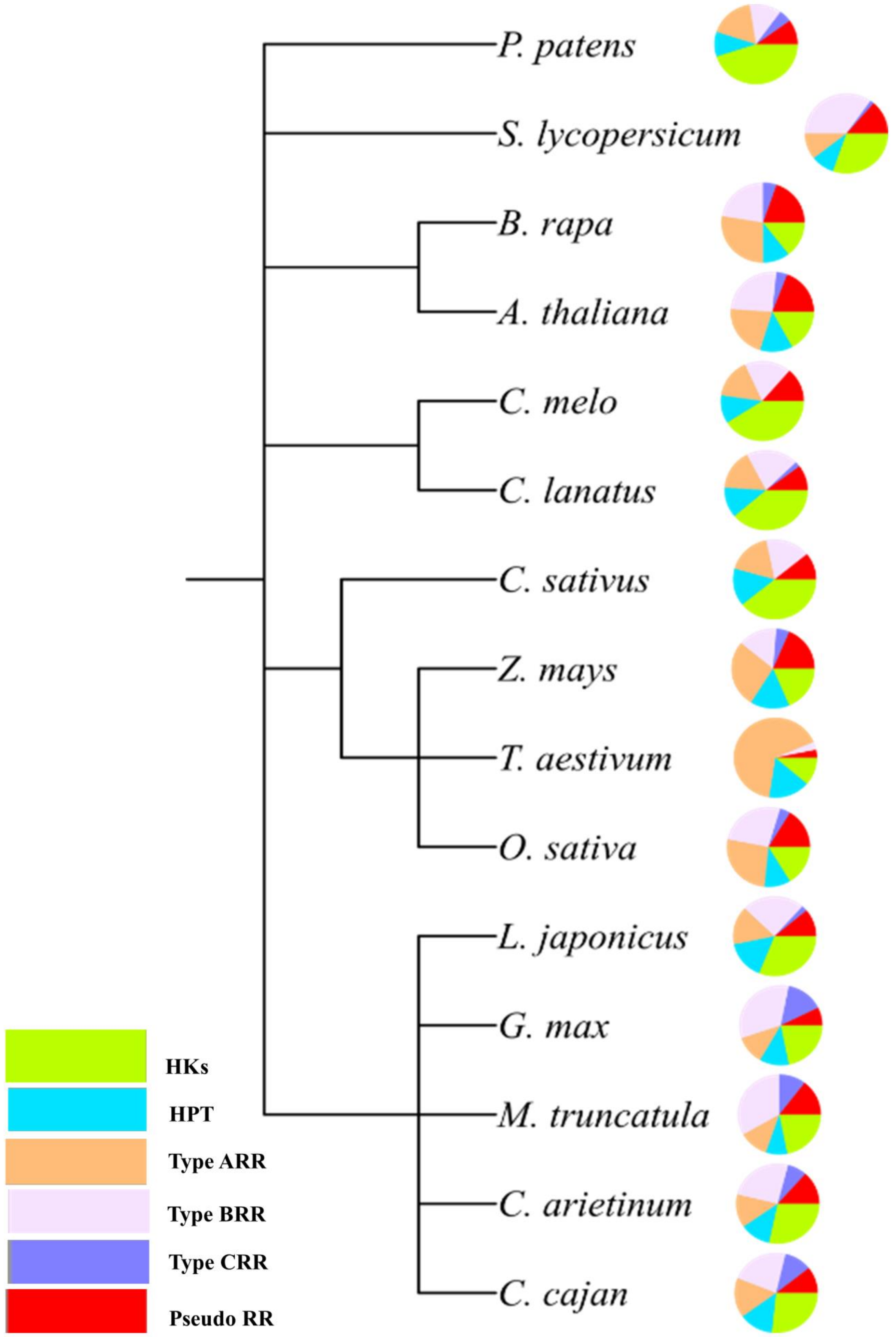

Supplemental Fig. 1. Phylogenetic classification of various crop species and distribution of TCS members

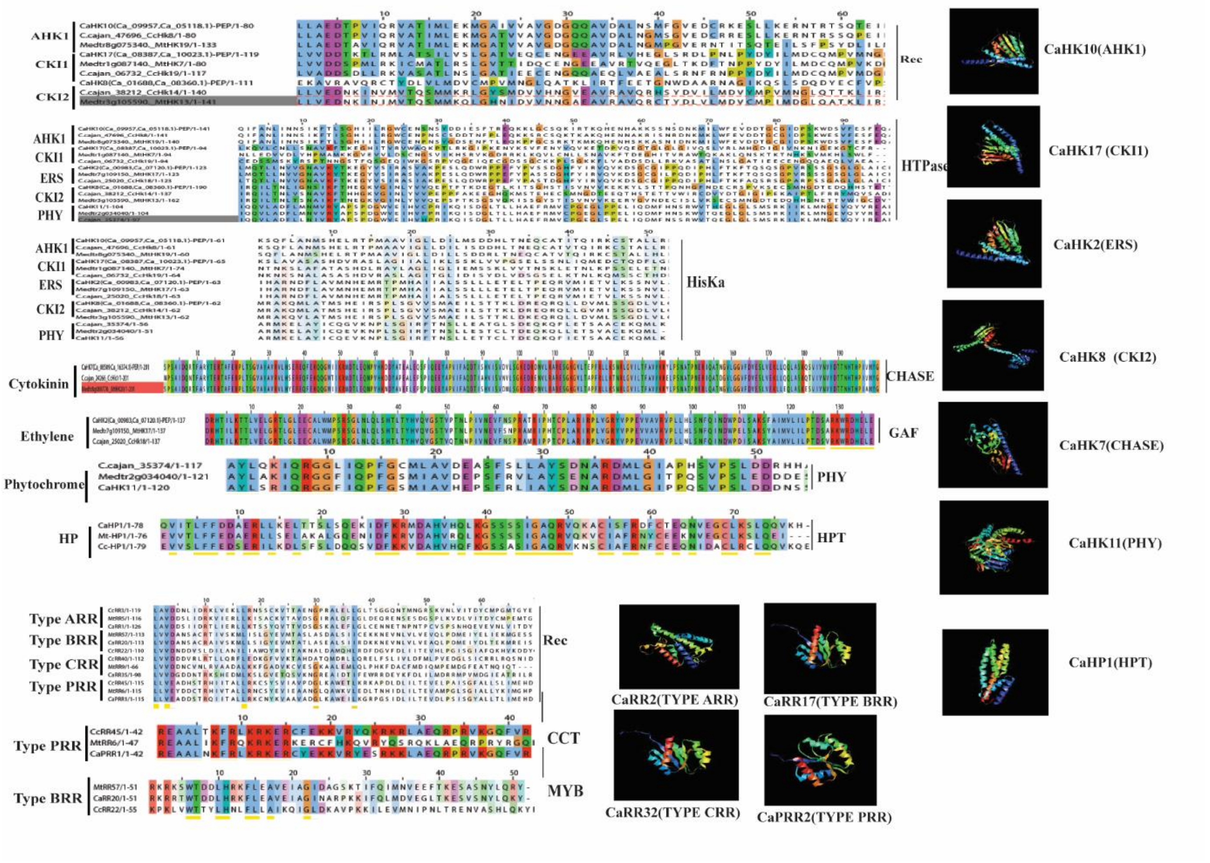

Supplemental Fig. 2. Domain arrangement and structural organisation. The multiple alignment of conserved domain and 3-dimensional structure of representative proteins from histidine kinase, histidine phosphotransferases and response regulator family.

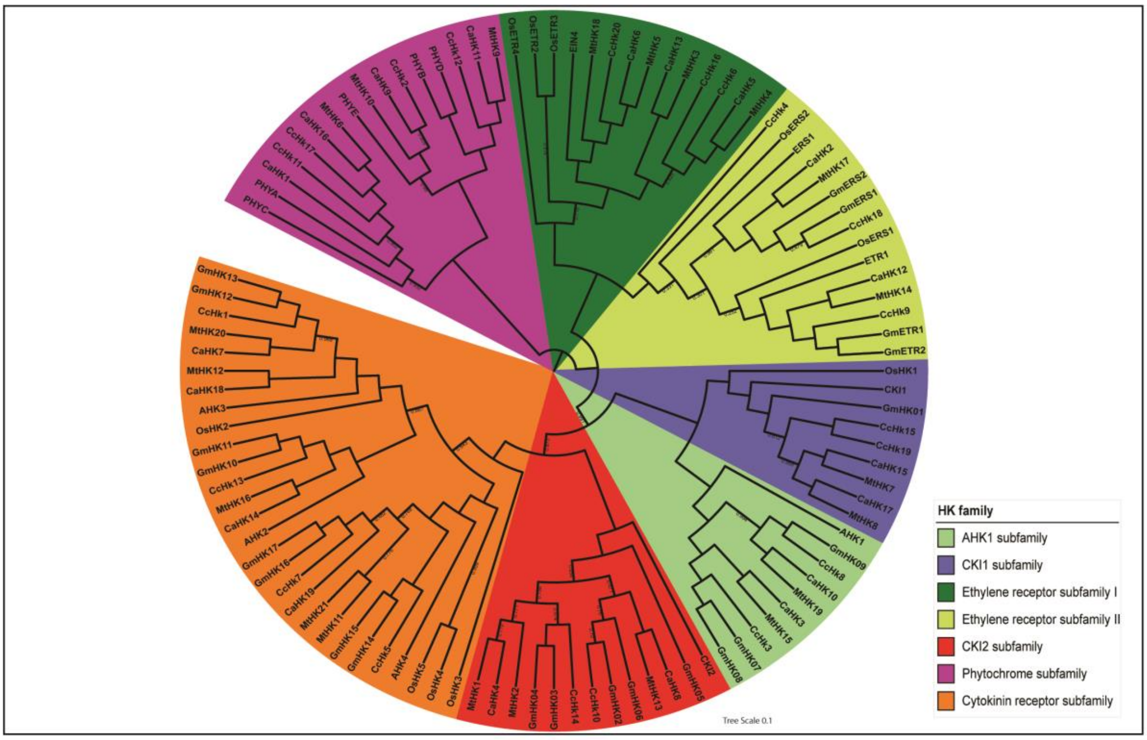

Supplemental Fig. 3. Phylogenetic analysis of HKs. Phylogenetic relationships of histidine kinase family proteins from chickpea, *Medicago, C. cajan,* soybean, rice and Arabidopsis. The bootstrap values are depicted as numerical values.

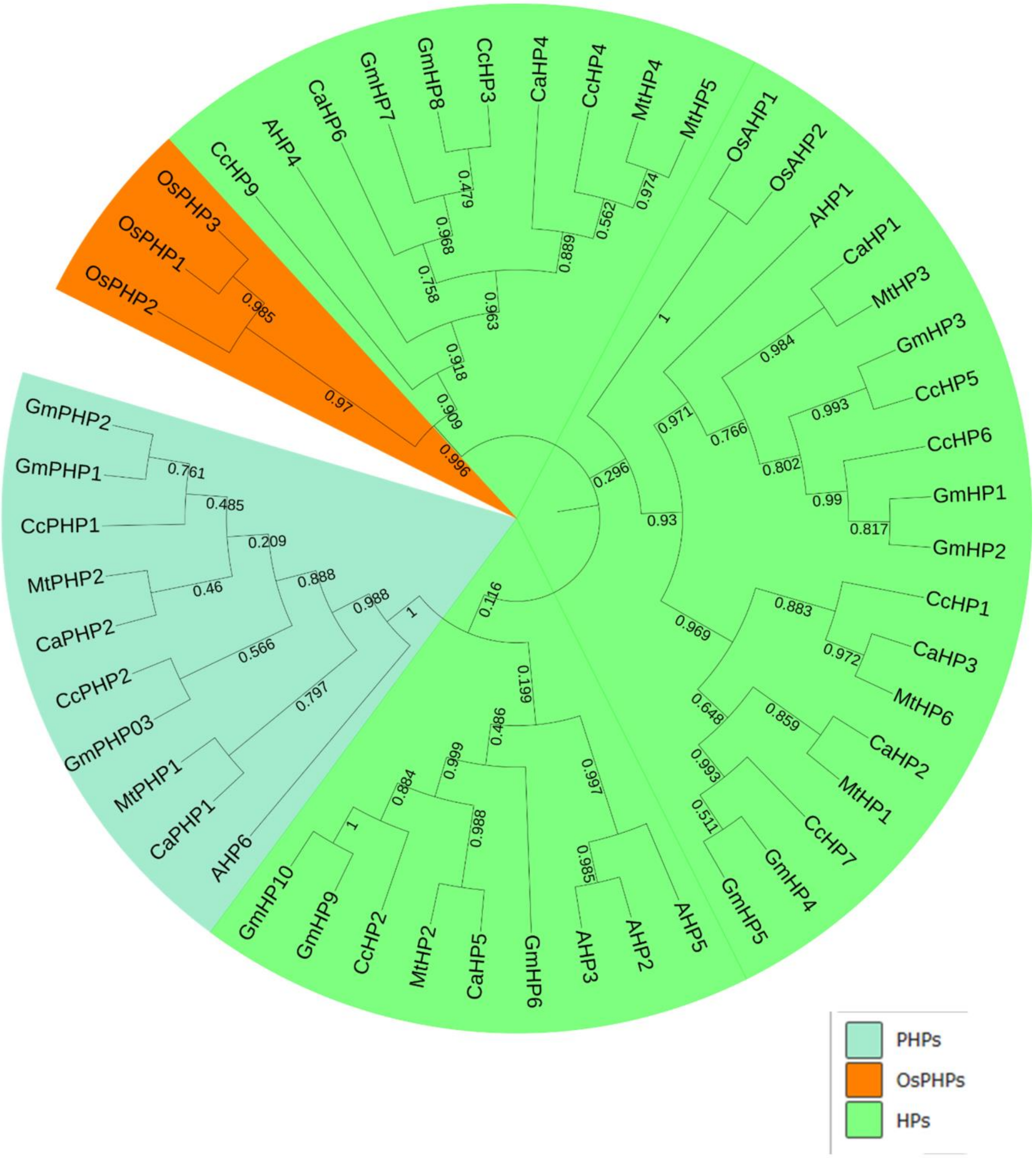

Supplemental Fig. 4. Phylogenetic analysis of HPs. The phylogenetic tree of histidine phosphotransferase and pseudo histidine phosphotransferase in different crops. The bootstrap values are depicted as values.

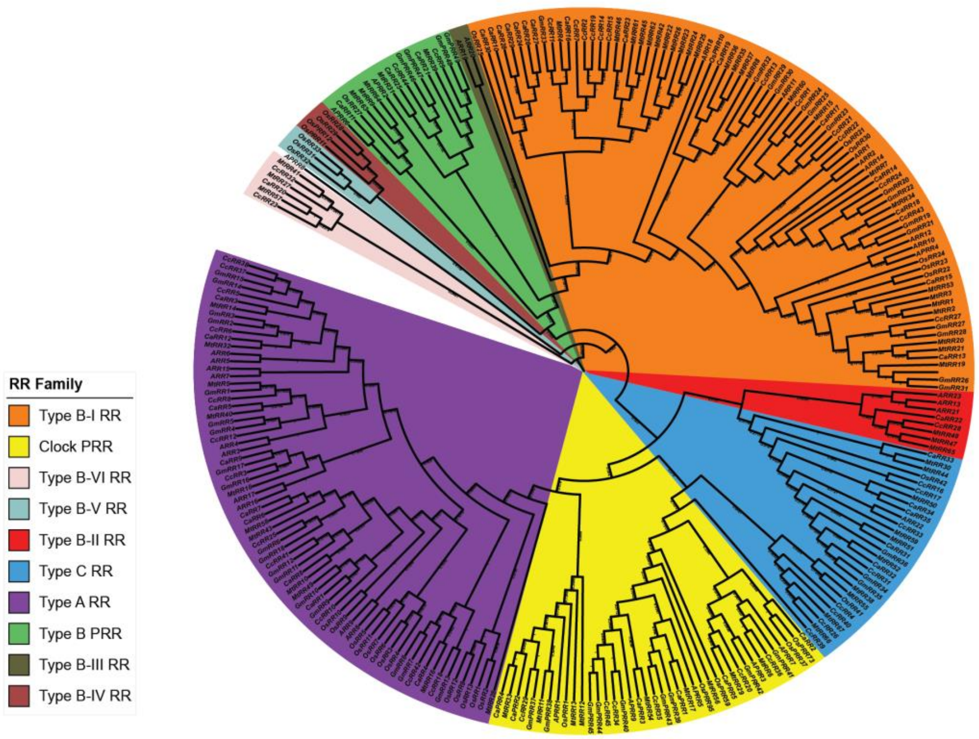

Supplemental Fig. 5. Phylogenetic analysis of RRs. The phylogenetic tree classified the response regulator family into TypeA RR, TypeB RR, TypeC RR and PseudoRR subfamily. The bootstrap values are depicted as numerical values.

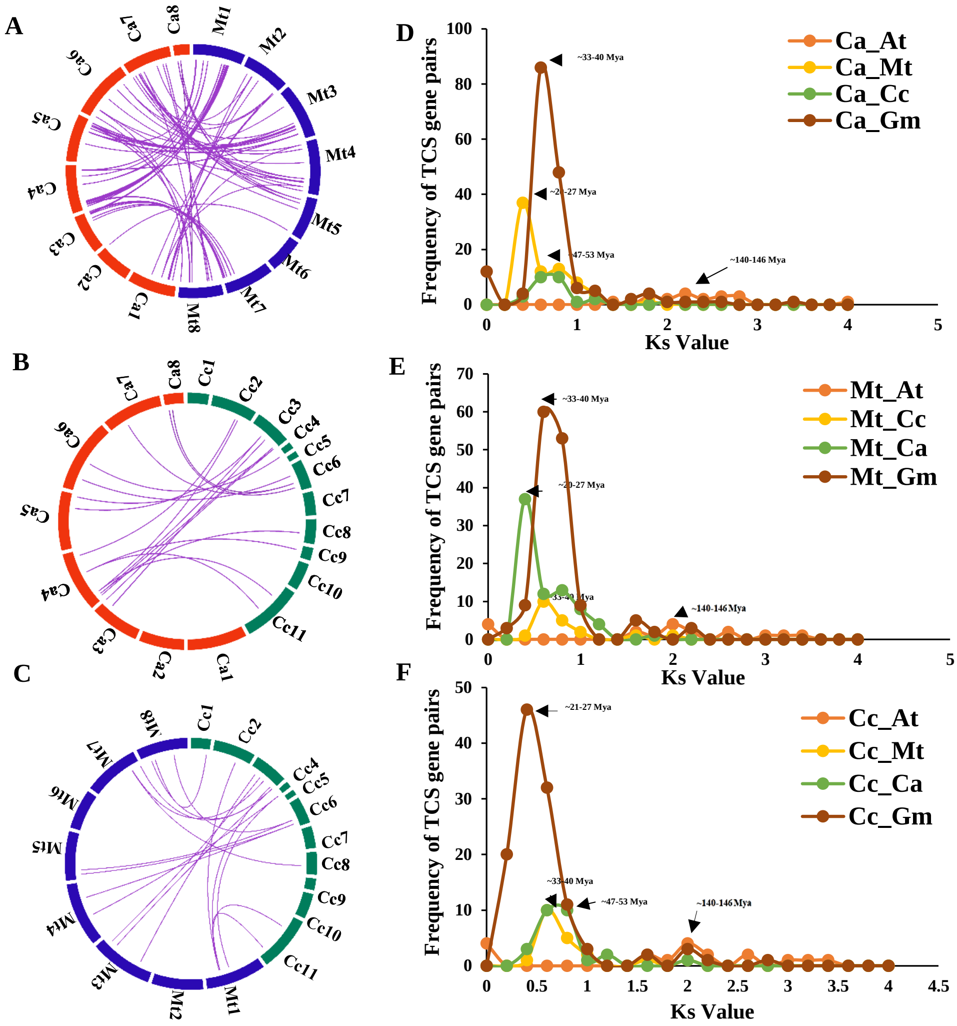

Supplemental Fig. 6. Synteny and collinearity analysis of TCS members among legumes. (A-C). Segmentally duplicated pairs in **(A)** chickpea-*Medicago* (Ca-Mt), **(B)** chickpea-pigeonpea (Ca-Cc) and **(C)** *Medicago*-pigeonpea (Mt-Cc) visualized by Circos (**D-F).** Graphs represent Ks values plotted against number of segmentally duplicated pairs for estimating time of divergence between the legumes and non-legumes (Arabidopsis), **(D)** chickpea, **(E)** *Medicago* and **(F)** *Cajanus cajan*. The Y-axis indicates the frequency of TCS gene pairs and the horizontal axis indicates the Ks values. Arrows are indicating the time of divergence in million year ago (Mya).

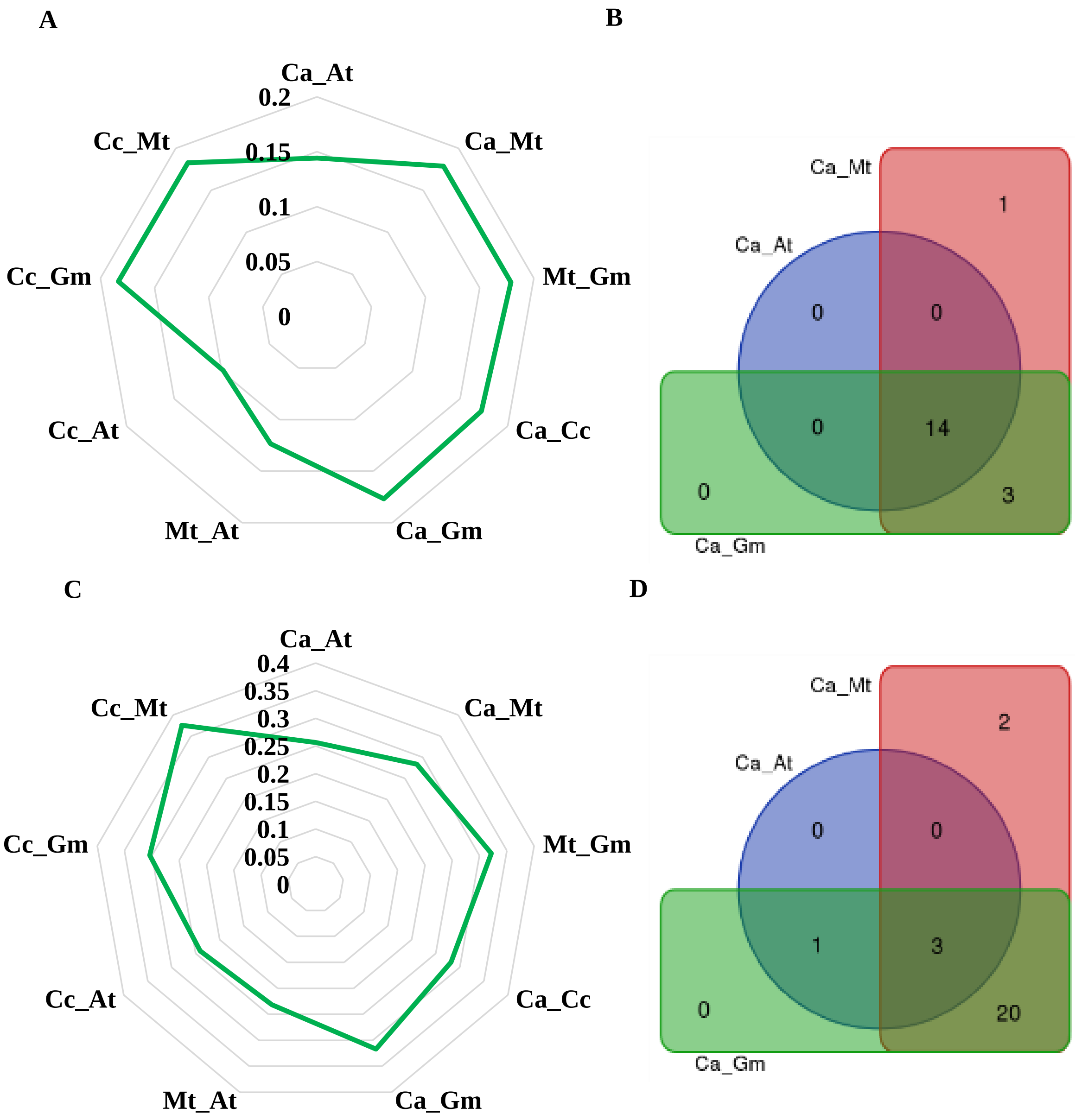

Supplemental Fig. 7. Evolutionary analysis of TCS members. (A-B). Spider web indicates pair-wise comparison of median values of Ka/Ks ratio of **(A)** HKs and **(B)** RRs **(C-D).** Venn diagram denotes the pair-wise comparison of number of **(C)** HKs and **(D)** RRs in in chickpea with Arabidopsis (Ca-At), chickpea with *M. truncatula* (Ca-Mt), chickpea with *Glycine max* (Ca-Gm), chickpea with Pigeonpea (Ca-Cc), *M. truncatula* with Arabidopsis (Mt-At), *M. truncatula* with *Glycine max* (Mt-Gm), Pigeonpea with *M. truncatula* (Cc-Mt), Pigeonpea with *Glycine max* (Cc-Gm) and), Pigeonpea with Arabidopsis (Cc-At).

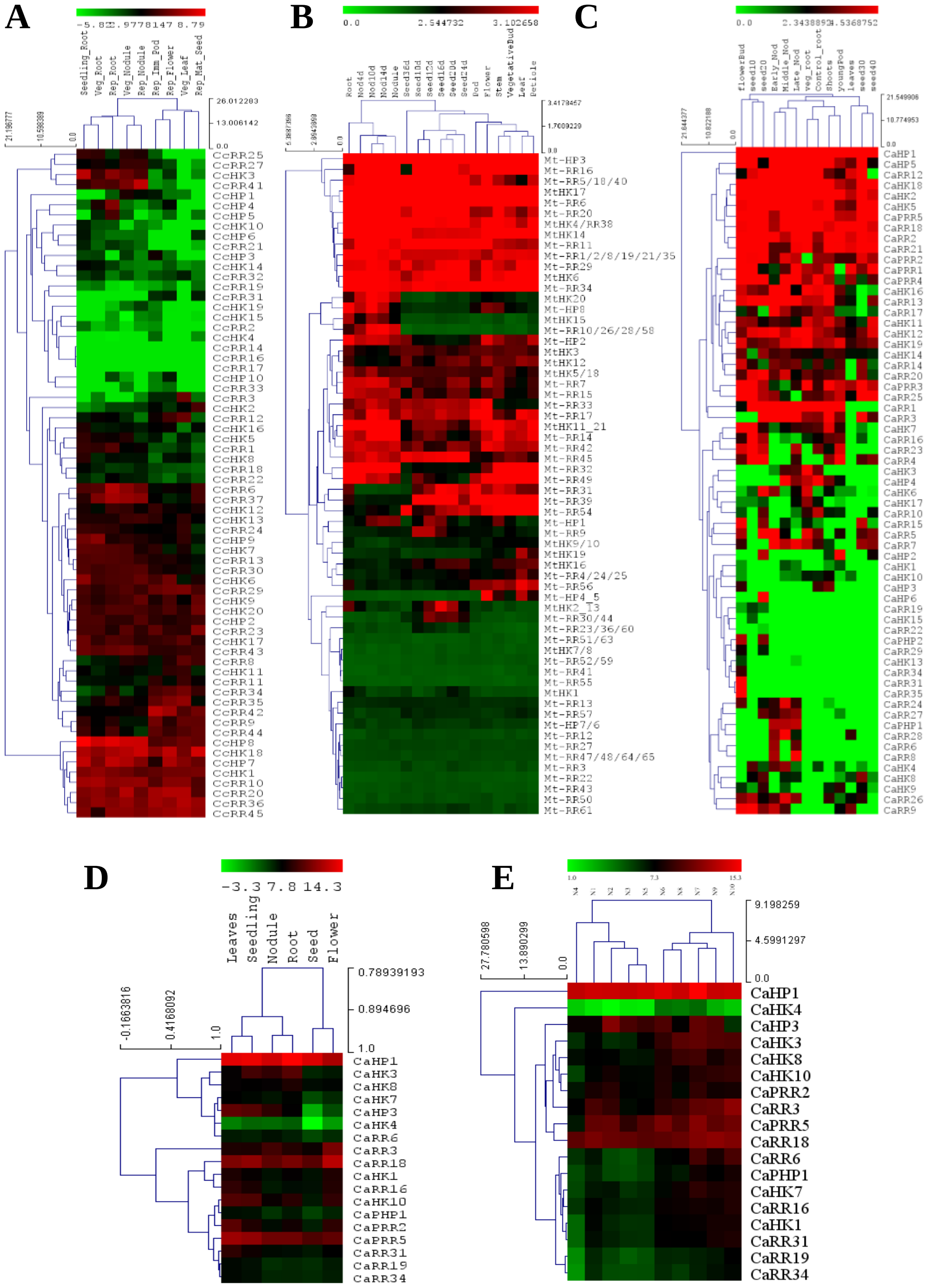

Supplemental Fig. 8. Digital Spatiotemporal and stress-responsive expression analyses of TCS members and qRT-PCR based validation. (A-C). The digital expression analysis in different tissues of **(A)** chickpea**, (B)** *Medicago* and **(C)** *C. cajan***. (D)** Heat map denotes the qRT-PCR based expression analysis of TCS members in different chickpea tissues**. (E)** Heat map denotes the qRT-PCR based expression analysis of TCS members in different nodule-specific stages (N1-1HPI, N2-3HPI, N3-6HPI, N4-12HPI, N5-24HPI, N6-3DPI, N7-7DPI, N8-14DPI, N9-21DPI, N10-28DPI). The experiments were carried out in triplicates and expressed as means ± S.E.

A

CaRR13 --- 616 AA (protein)
1852 nt (CDS)

B

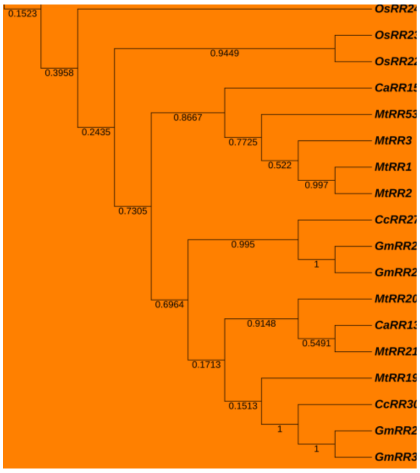

C

>CaRR13(Ca_01726,Ca_14780.1)-PEP
MDDPSDRFPIGMRVLAVDDDPTCLFVLETLLRRCQYHVTTTSQAIKALAMLRENKD
KFDLVISDVHMPDMDGFKLLELVGLEMDLPVIMLSAYGDTKLVMKGISHGACDYLL
KPVRIEELKNIWQHVIRRKKSDPKEKNKTSNRDKTTSDSGEENGKLNKKRKDQDED
EDEDKENDHDNEDPSAQKKPRVVWSVELHRKFVDAVNRLGIDKAVPKKILDMMN
VENITRENKYRLYLKRISCVANQQASMVAAFGSADQSYLRMAGSGHFHNNAFRSF
SSSGFISSLNTPAGLNGHGFPPSGILQLGQSQNLNNSSNDQLKFQSAITPVNQNVLH
GTPMSIGFDHSQNSKGVLSVQNLTTDDVKTTFPIPDQRLRLATSSSYPPSLGITNNAS
SSSGASRHSEFPFSMIDQSRHSDNWSSAVQLSGIQTNSFPSSECFRQTAIPPSDNMA
SLPLQGAYLSGQSIASLCSQSHGSLADMHSQGAFFTNPPEYGNSNLPFQGWEDHN
QDAIYHSNVTCGSTNSPFSGAVVPSGLTTTNSALHRNLDTRFCDDPIQMKHAGFGE
LVECSSSRQPRANVVSQQKFSNNLGSLEYLVSSTMEQEQDKMKLLNGDLICDNYSL

Rec Domain   Myb Domain   Nuclear localization signal

D

Sequences producing significant alignments   Download   Manage Columns   Show 100

select all 3 sequences selected   Graphics   Distance tree of results   Multiple alignment

	Description	Max Score	Total Score	Query Cover	E value	Per. Ident	Accession
☑	Mt-RR21	964	964	99%	0.0	77.81%	Query_10007
☑	Mt-RR20(Medtr3g1026001)	216	340	48%	1e-70	81.95%	Query_10009
☑	DNA binding site	87.0	106	12%	3e-25	67.80%	Query_10008

Supplemental Fig. 9. CaRR13 sequence, domain and homology. (**A**) The nucleotide and the amino acid length of the *CaRR13* gene and protein. (**B**) The Type-B RR clade representing clustering of CaRR13 in a legume specific clade. (**C**) Marked functional domains and DNA binding segment of CaRR13 protein. (**D**) Percent identity of CaRR13 protein with *Medicago* homologs MtRR20 and 21.
