# Supplementary material for: Evolutionary and functional analysis of two‐component system in chickpea reveals CaRR13, a TypeB RR, as positive regulator of symbiosis
Source: Plant Biotechnol J. 2021 Sep 16;19(12):2415–27. doi: 10.1111/pbi.13649 (PMC8633487; doi:10.1111/pbi.13649)
Supplement: Supplementary file 2 — Table S1 List of TCS genes with their associated domain in chickpea, Medicago and C. cajan. Table S2 Distribution of TCS genes across various classes in the 3 legumes analysed in this study (marked by *) and other plants. Table S3 (A) A list of paralogs in chickpea, Medicago and C. cajan genome. (B) A list of orthologs pairs in chickpea with Medicago, Cajanus and Glycine. (C) A list of orthologs pairs in Medicago ‐Glycine, Medicago‐Cajanus and Cajanus‐Glycine. Table S4 Pairwise comparison of segmentally duplicated TCS pairs present on chromosome in legumes. Table S5 A list of orthologous pairs in legumes with Arabidopsis. Table S6 The ratio of total genes and TCS gene in Medicago‐C. cajan, Medicago‐chickpea and chickpea‐C. cajan. [file PBI-19-2415-s002.pdf]

Supplemental Table 1: List of TCS genes with their associated domain in chickpea, *Medicago* and *C. cajan*

TCS genes	locus id	Domain						
Cicer arietinum			*Medicago truncatula*			*Cajanus cajan*		
CaHK1	Ca_01069,Ca_07198.1	HATPase_c, PAS, PHY, GAF, HisKA	MtHK1	Medtr1g013360	REC, HATPase_c, HisKA, PAS_4, PRK11107	CcHk1	C.cajan_24266	REC, HATPase_c, HisKA, PRTases_typeI, CHASE
CaHK2	Ca_00983,Ca_07120.1	HATPase_c, HisKA, GAF	MtHK10	Medtr2g049520	HATPase_c, HisKA, PAS, PHY, PAS_2, GAF	CcHk10	C.cajan_09255	HATPase_c, REC, HisKA
CaHK3	Ca_01956,Ca_23349.1	REC, HATPase_c, HisKA	MtHK11	Medtr2g067240	HisKA, CHASE	CcHk11	C.cajan_10650	HATPase_c, PAS, PHY, PAS_2, GAF, HisKA, BaeS
CaHK4	Ca_09112,Ca_09608.1	REC, HATPase_c, HisKA	MtHK12	Medtr3g085130	REC, HATPase_c, HisKA, PRTases_typeI, CHASE	CcHk12	C.cajan_35374	HATPase_c, PAS, PHY, PAS_2, GAF, HisKA, BaeS
CaHK5	Ca_12071,Ca_08578.1	REC, HisKA, GAF	MtHK13	Medtr3g105590	REC, HATPase_c, HisKA, PAS_4	CcHk13	C.cajan_27186	REC, HATPase_c, HisKA, CHASE
CaHK6	Ca_03800,Ca_08907.1	REC, HisKA, GAF	MtHK14	Medtr4g031150	HATPase_c, REC, HisKA, GAF, BaeS, OmpR	CcHk14	C.cajan_38212	HATPase_c, REC, HisKA, pfam08448
CaHK7	Ca_08509,Ca_16374.1	REC, HATPase_c, HisKA, CHASE	MtHK15	Medtr5g022470	REC, HATPase_c, HisKA, PRK11107	CcHk15	C.cajan_01249	HATPase_c, REC, HisKA
CaHK8	Ca_01688,Ca_08360.1	HATPase_c, REC, HisKA, FlgN	MtHK16	Medtr5g097410	REC, HATPase_c, HisKA, CHASE, PRK11107	CcHk16	C.cajan_02781	REC, GAF, HATPase_c
CaHK9	Ca_22572,Ca_01534.1	HATPase_c, HisKA, PAS, PHY, PAS_2, GAF	MtHK17	Medtr7g109150	HATPase_c, HisKA, GAF, BaeS	CcHk17	C.cajan_03784	HATPase_c, PAS, PHY, PAS_2, GAF, HisKA
CaHK10	Ca_09957,Ca_05118.1	REC, HATPase_c, HisKA	MtHK18	Medtr7g116330	REC, HisKA, GAF	CcHk18	C.cajan_25020	HATPase_c, GAF, HisKA, BaeS
CaHK11	Ca_08096,Ca_01970.1	HATPase_c, HisKA, PAS, PHY, PAS_2, GAF	MtHK19	Medtr8g075340	REC, HATPase_c, HisKA, PRK11107	CcHk19	C.cajan_06732	HATPase_c, REC, HisKA
CaHK12	Ca_25958,Ca_26097.1	HATPase_c, REC, HisKA, GAF	MtHK2	Medtr1g014670	HisKA, PAS_4	CcHk2	C.cajan_11391	HATPase_c, PAS, PHY, PAS_2, GAF, BaeS
CaHK13	Ca_21299,Ca_11555.1	HisKA, GAF	MtHK20	Medtr8g080770	REC, HATPase_c, HisKA, PRTases_typeI, CHASE	CcHk20	C.cajan_07004	REC, GAF
CaHK14	Ca_09784,Ca_03183.1	REC, HATPase_c, HisKA, CHASE, DUF4070	MtHK21	Medtr8g106150	REC, HATPase_c, HisKA, CHASE, CheY	CcHk3	C.cajan_12521	HATPase_c, REC, HisKA
CaHK15	Ca_03509,Ca_09832.1	REC, HATPase_c, HisKA	MtHK3	Medtr1g044210	REC, GAF, HisKA	CcHk4	C.cajan_14834	HATPase_c, GAF, HisKA
CaHK16	Ca_03582,Ca_08187.1	HATPase_c, PAS, PHY, PAS_2, GAF, HisKA	MtHK4	Medtr1g073840	REC, GAF, HisKA	CcHk5	C.cajan_29353	HATPase_c, REC, HisKA, CHASE
CaHK17	Ca_08387,Ca_10023.1	REC, HATPase_c, HisKA	MtHK5	Medtr1g079790	REC, GAF, HisKA	CcHk6	C.cajan_22542	REC, GAF
CaHK18	Ca_11358,Ca_13609.1	REC, HATPase_c, PRTases_typeI, HisKA, CHASE	MtHK6	Medtr1g085160	HATPase_c, PAS, PHY, PAS_2, GAF, HisKA	CcHk7	C.cajan_15537	HATPase_c, REC, HisKA, CHASE
CaHK19	Ca_10390,Ca_15433.1	REC, HATPase_c, HisKA, REC, CHASE	MtHK7	Medtr1g087140	REC, GAF, HisKA, OmpR	CcHk8	C.cajan_47696	HATPase_c, REC, HisKA
CaHP1	Ca_00554,Ca_02373.1	HPT	MtHK8	Medtr1g090850	REC, GAF, HATPase_c, OmpR, HisKA	CcHk9	C.cajan_38982	HATPase_c, REC, HisKA, GAF, BaeS
CaHP2	Ca_03717,Ca_09237.1	HPT	MtHK9	Medtr2g034040	HATPase_c, PAS, PHY, PAS_2, GAF, HisKA, BaeS	Cc-HP1	C.cajan35868	Hpt
CaHP3	Ca_01193,Ca_27908.1	HPT	Mt-HP1	Medtr1g082290	Hpt	Cc-HP2	C.cajan10483	Hpt
CaHP4	Ca_02785,Ca_00222.1	HPT	Mt-HP2	Medtr1g089130	Hpt	CcPHP1	C.cajan00223	Hpt
CaHP5	Ca_08308,Ca_09933.1	HPT	Mt-HP3	Medtr2g020770	Hpt	Cc-HP4	C.cajan00306	Hpt
CaHP6	Ca_10140,Ca_24173.1	HPT	Mt-HP4	Medtr2g100880	Hpt	Cc-PHP2	C.cajan00431	Hpt
CaPHP1	Ca_02886,Ca_00159.1	HPT	Mt-HP5	Medtr2g100900	Hpt	Cc-HP5	C.cajan02977	Hpt
CaPHP2	Ca_19186,Ca_19712.1	HPT	Mt-PHP1	Medtr2g103870	Hpt	Cc-HP6	C.cajan04331	Hpt
CaRR1	Ca_07192,Ca_06912.1	REC	Mt-PHP2	Medtr4g010160	Hpt	Cc-HP7	C.cajan05249	Hpt
CaRR2	Ca_14042,Ca_11231.1	REC	Mt-HP6	Medtr7g114020	Hpt	Cc-HP8	C.cajan06887	Hpt
CaRR3	Ca_07527,Ca_13482.1	REC	MtRR1	Medtr1g013160	REC, SANT, OmpR	Cc-HP9	C.cajan36466	Hpt
CaRR4	Ca_12649,Ca_13873.1|	REC	MtRR2	Medtr1g013170	myb_SHAQKYF, REC, SANT	CcRR1	C.cajan_24323	myb_SHAQKYF, REC, SANT
CaRR5	Ca_23422	REC	MtRR3	Medtr1g013180	myb_SHAQKYF, REC, SANT, RNase_H_like superfamily	CcRR2	C.cajan_37366	myb_SHAQKYF, REC, SANT
CaRR6	Ca_17994,Ca_11981.1	REC	MtRR4	Medtr1g032570	myb_SHAQKYF, REC, SANT	CcRR3	C.cajan_23647	REC
CaRR7	Ca_25617,Ca_09364.1	REC	MtRR5	Medtr1g049100	REC	CcRR4	C.cajan_46866	REC
CaRR8	Ca_24591,Ca_17810.1|	REC	MtRR6	Medtr1g067110	REC, CCT	CcRR5	C.cajan_35128	REC
CaRR9	Ca_04102,Ca_14081.1	REC	MtRR7	Medtr2g034960	myb_SHAQKYF, REC, SANT	CcRR6	C.cajan_34072	REC
CaRR10	Ca_24514,Ca_17837.1	REC, SANT, RAMP_I_III	MtRR8	Medtr2g450070	myb_SHAQKYF, REC, SANT	CcRR7	C.cajan_11822	myb_SHAQKYF, REC, SANT
CaRR11	Ca_05544,Ca_11868.1	SANT, REC	MtRR9	Medtr2g050020	CheY, REC	CcRR8	C.cajan_11838	REC
CaRR12	Ca_15303,Ca_21195.1	REC	MtRR10	Medtr3g015490	REC	CcRR9	C.cajan_12949	myb_SHAQKYF, REC, SANT
CaRR13	Ca_01726,Ca_14780.1	REC, SANT	MtRR11	Medtr3g037390	REC, CCT, OmpR	CcRR10	C.cajan_30148	REC
CaRR14	Ca_14780,Ca_18993.1|	REC, SANT	MtRR12	Medtr3g037400	MeCP2_MBD, REC, OmpR	CcRR11	C.cajan_29328	myb_SHAQKYF, REC, SANT
CaRR15	Ca_09080,Ca_09615.1	REC, SANT	MtRR13	Medtr3g037450	REC	CcRR12	C.cajan_40143	REC
CaRR16	Ca_15151,Ca_29339.1	REC, SANT	MtRR14	Medtr3g078613	REC	CcRR13	C.cajan_22328	myb_SHAQKYF, REC, SANT
CaRR17	Ca_11309,Ca_13743.1	REC, SANT	MtRR15	Medtr3g086100	REC, myb_SHAQKYF, SANT	CcRR14	C.cajan_15557	myb_SHAQKYF, REC, SANT
CaRR18	Ca_02989,Ca_19524.1	REC, SANT	MtRR16	Medtr3g088630	REC, PLN03029	CcRR15	C.cajan_15558	myb_SHAQKYF, REC, SANT
CaRR19	Ca_03351	REC, SANT	MtRR17	Medtr3g092780	REC, CCT, OmpR	CcRR16	C.cajan_16448	REC
CaRR20	Ca_16401,Ca_30098.1	REC, SANT	MtRR18	Medtr3g093860	REC	CcRR17	C.cajan_16449	REC
CaRR21	Ca_02285,Ca_22692.1	REC, SANT	MtRR19	Medtr3g102590	REC, SANT	CcRR18	C.cajan_29859	REC
CaRR22	Ca_20899,Ca_09361.1	REC, SANT	MtRR20	Medtr3g102600	myb_SHAQKYF, REC, SANT	CcRR19	C.cajan_44120	myb_SHAQKYF, REC, SANT
CaRR23	Ca_23740	REC, SANT	MtRR21	Medtr3g106220	myb_SHAQKYF, REC, SANT	CcRR20	C.cajan_21442	REC, CCT
CaRR24	Ca_21939	REC, SANT	MtRR22	Medtr4g021760	myb_SHAQKYF, REC, SANT	CcRR21	C.cajan_19355	REC
CaRR25	Ca_12825,Ca_20933.1	REC, SANT	MtRR23	Medtr4g021790	myb_SHAQKYF, REC, SANT	CcRR22	C.cajan_19358	myb_SHAQKYF, REC, SANT
CaRR26	Ca_24482,Ca_26382.1	REC, SANT	MtRR24	Medtr4g021845	myb_SHAQKYF, REC, SANT	CcRR23	C.cajan_20619	REC
CaRR27	Ca_26462	REC, SANT	MtRR25	Medtr4g021855	myb_SHAQKYF, REC, SANT	CcRR24	C.cajan_29495	myb_SHAQKYF, REC, SANT
CaRR28	Ca_24473	REC	MtRR26	Medtr4g023980	REC, SANT	CcRR25	C.cajan_41309	REC
CaRR29	Ca_26464	REC	MtRR27	Medtr4g028380	REC	CcRR26	C.cajan_47820	REC
CaRR30	Ca_27875,Ca_17948.1	REC	MtRR28	Medtr4g051330	REC	CcRR27	C.cajan_45197	myb_SHAQKYF, REC, SANT
CaRR31	Ca_12277,Ca_07427.1	REC	MtRR29	Medtr4g061360	REC, CCT	CcRR28	C.cajan_46352	REC
CaRR32	Ca_12296,Ca_07445.1	REC	MtRR30	Medtr4g071650	REC	CcRR29	C.cajan_08715	REC, CCT, OmpR
CaRR33	Ca_20387	REC	MtRR31	Medtr4g098870	myb_SHAQKYF, REC, SANT	CcRR30	C.cajan_09230	myb_SHAQKYF, REC, SANT
CaRR34	Ca_12286,Ca_07435.1	REC	MtRR32	Medtr4g106590	myb_SHAQKYF, REC, SANT	CcRR31	C.cajan_09726	REC
CaRR35	Ca_27703,Ca_07435.1	REC	MtRR33	Medtr4g108880	REC, CCT, OmpR	CcRR32	C.cajan_10804	REC, GAF
CaPRR1	Ca_04056,Ca_14266.1	REC, CCT	MtRR34	Medtr4g121020	myb_SHAQKYF, REC, SANT	CcRR33	C.cajan_10814	REC
CaPRR2	Ca_18243,Ca_12864.1	REC, CCT	MtRR35	Medtr4g131570	myb_SHAQKYF, REC, SANT	CcRR34	C.cajan_10962	REC, CCT
CaPRR3	Ca_01359,Ca_07096.1	REC, CCT	MtRR36	Medtr4g131580	myb_SHAQKYF, REC, SANT,OmpR	CcRR35	C.cajan_39146	REC, CCT, OmpR
CaPRR4	Ca_13165,Ca_21411.1	REC, CCT	MtRR37	Medtr4g131600	myb_SHAQKYF, REC, SANT,OmpR	CcRR36	C.cajan_41432	REC, CCT
CaPRR5	Ca_14621,Ca_18487.1	PKc_like, REC, CCT	MtRR38	Medtr4g134880	REC, OmpR	CcRR37	C.cajan_31558	REC
			MtRR39	Medtr5g014040	myb_SHAQKYF, REC, SANT	CcRR38	C.cajan_40453	REC
			MtRR40	Medtr5g036480	REC	CcRR39	C.cajan_48325	REC, OmpR
			MtRR41	Medtr5g044100	REC	CcRR40	C.cajan_48336	REC, OmpR
			MtRR42	Medtr5g055260	REC, SANT, OmpR	CcRR41	C.cajan_01143	REC
			MtRR43	Medtr6g007460	REC	CcRR42	C.cajan_03110	REC
			MtRR44	Medtr6g008860	REC	CcRR43	C.cajan_04108	myb_SHAQKYF, REC, SANT
			MtRR45	Medtr6g016850	REC, OmpR	CcRR44	C.cajan_40483	myb_SHAQKYF, REC, SANT
			MtRR46	Medtr6g045327	REC, SANT	CcRR45	C.cajan_39868	REC, CCT
			MtRR47	Medtr6g088950	REC			
			MtRR48	Medtr7g026400	myb_SHAQKYF, REC, SANT			
			MtRR49	Medtr7g490310	REC, PLN03029			
			MtRR50	Medtr7g098860	REC			
			MtRR51	Medtr7g098910	REC			
			MtRR52	Medtr7g099500	REC			
			MtRR53	Medtr7g117705	myb_SHAQKYF, REC, SANT			
			MtRR54	Medtr7g118260	REC, CCT			
			MtRR55	Medtr8g019680	REC			
			MtRR56	Medtr8g024260	REC, CCT			
			MtRR57	Medtr8g032710	REC, SANT			
			MtRR58	Medtr8g038620	REC, PLN03029			
			MtRR59	Medtr8g063580	REC			
			MtRR60	Medtr8g079940	myb_SHAQKYF, REC, SANT, PRK10161			
			MtRR61	Medtr8g093040	REC, OmpR			
			MtRR62	Medtr8g105600	REC, SANT, OmpR			
			MtRR63	Medtr0022s0450	REC superfamily			
			MtRR64	Medtr0054s0010	REC superfamily			
			MtRR65	Medtr0450s0040	myb_SHAQKYF, REC, SANT			
			MtRR66	Medtr1727s0010	REC, OmpR			
			MtRR67	Medtr1874s0010	REC, trans_reg_C, HTH superfamily, OmpR			

Supplemental Table 2: Distribution of TCS genes across various classes in the 3 legumes analysed in this study (marked by *) and other plants

Species	HK	HPt	Type-A RR	Type-B RR	Type-C RR	Pseudo-RR	Total
*Cicer arietinum**	19	8	10	16	9	5	67
*Medicago truncatula**	21	8	11	35	12	9	96
*Cajanus cajan**	20	10	12	16	11	6	75
Arabidopsis thaliana	8	6	10	12	2	9	47
Oryza sativa	8	5	13	13	2	8	49
Lotus japonicus	14	7	7	11	1	5	45
Glycine max	21	13	18	15	3	13	83
Zea mays	11	9	16	9	3	11	59
Physcomitrella patens	18	3	7	5	2	4	39
Brassica rapa	11	8	21	17	4	15	76
Solanum lycopersicum	20	6	7	23	1	8	65
Cucumis melo	18	5	7	8	0	6	44
Triticum aestivum	7	10	41	2	0	2	62
Cucumis sativus	18	7	8	8	0	5	46
Citrullus lanatus	19	6	8	10	1	5	49

Further distribution of TCS members in the 3 legumes

	Cytokinin rec	Ethylene rece	Phytochrome receptor	AHK1	CKI1	CKI2	HP	PHP	Type-A RR	Type-B RR	Type-C RR	Pseudo-RR	Total
*Cicer arietinum**	4	5	4	2	2	2	6	2	10	16	9	5	67
*Medicago truncatula**	5	6	3	2	2	3	6	2	11	35	12	9	96
*Cajanus cajan**	4	6	4	2	2	2	8	2	12	16	11	6	75

Supplemental Table 3A: A list of paralogs in chickpea, *Medicago* and *C. cajan* genome

Ca_TCS	Ca_TCS	Ka	Ks	Ka/Ks
CaHK1	CaHK16	0.1177	0.6406	0.183733999
CaHK10	CaHK3	0.1375	0.7002	0.196372465
CaHK5	CaHK6	0.3375	2.1854	0.154433971
CaHK5	CaHK13	0.1828	0.9816	0.186226569
CaHK7	CaHK18	0.1013	0.6644	0.152468393
CaHK8	CaHK4	0.1039	0.5556	0.18700504
CaHP1	CaHP5	0.2888	0	
CaHP3	CaHP2	0.1792	0.6691	0.267822448
CaHP4	CaHP6	0.1243	0.79	0.157341772
CaPHP1	CaPHP2	0.0928	0.9633	0.096335513
CaPRR3	CaPRR1	0.5458	1.5912	0.343011564
CaRR12	CaRR3	0.1686	0.7857	0.21458572
CaRR13	CaRR15	0.3612	1.0141	0.356177892
CaRR18	CaRR14	0.2016	0.8833	0.228235028
CaRR25	CaRR21	0.1572	0.5749	0.273438859

Mt_TCS	Mt_TCS	Ka	Ks	Ka/Ks
MtHK1	MtHK13	0.1494	0.7407	0.201701094
MtHK12	MtHK20	0.1138	0.7157	0.15900517
MtHK15	MtHK19	0.1476	0.8078	0.182718495
MtHK3	MtHK4	0.169	0.8182	0.206550966
MtHK5	MtHK18	0.1745	0.7424	0.235048491
Mt-HP1	Mt-HP8	0.2152	0.6662	0.323026118
Mt-HP2	Mt-HP3	0.325	0	
Mt-HP6	Mt-HP7	0.0797	1.1673	0.068277221
MtRR1	MtRR36	0.4126	1.5367	0.26849743
MtRR1	MtRR21	0.2134	0.5977	0.357035302
MtRR10	MtRR58	0.172	0.5771	0.298041934
MtRR11	MtRR33	0.1866	0.5605	0.332917038
MtRR14	MtRR32	0.1597	0.9564	0.166980343
MtRR15	MtRR60	0.2222	0.609	0.364860427
MtRR17	MtRR56	0.7303	1.6914	0.431772496
MtRR17	MtRR54	0.5935	1.5411	0.385114529
MtRR2	MtRR35	0.5972	1.6431	0.363459315
MtRR31	MtRR39	0.1808	0.5867	0.308164309
MtRR32	MtRR40	0.3523	1.7214	0.204658998
MtRR54	MtRR56	0.4135	0.8055	0.513345748
MtRR6	MtRR29	0.5169	2.1639	0.238874255
MtRR7	MtRR34	0.1866	0.8556	0.218092567

Cc_TCS	Cc_TCS	Ka	Ks	Ka/Ks
CcHk20	CcRR32	0.1363	0.6408	0.212702871
CcHk20	CcHk6	0.32	1.7609	0.181725254
CcHP8	CcHP2	0.2964	-1	-0.2964
CcRR21	CcRR1	0.0836	0.5071	0.164859002
CcRR37	CcRR6	0.2176	0.7925	0.274574132
CcRR8	CcRR12	0.1039	0.7482	0.138866613

Mean of Ka and Ks values

	Mean Ka_values HKs	0.16345
Ca	Mean Ka_values RRs	0.28688
	Mean Ka_values HKs	0.15086
Mt	Mean Ka_values RRs	0.352686
	Mean Ks_values HKs	0.954633
Ca	Mean Ks_values RRs	0.96984
	Mean Ks_values HKs	0.76496
Mt	Mean Ks_values RRs	1.131864

Ratio of Ka and Ks values RRs Vs HKs

Ca_Ka ratios	
Ka_RRs/Ka_HKs	1.755154
Mt_Ka ratios	
Mt	
Ka_RRs/Ka_HKs	2.337835
Ca_Ks ratios	
Ca	
Ks_RRs/Ks_HKs	1.015929
Mt_Ks ratios	
Mt	
Ks_RRs/Ks_HKs	1.479639

Supplemental Table 3B: A list of orthologs pairs in chickpea with *Medicago* , *Cajanus* and *Glycine*

Ca_TCS	Mt_TCS	Ka	Ks
CaHK1	MtHK6	0.1206	0.6826
CaHK10	MtHK19	0.0634	0.2807
CaHK10	MtHK15	0.1342	0.7453
CaHK11	MtHK9	0.0435	0.319
CaHK12	MtHK17	0.0125	0.2973
CaHK13	MtHK3	0.146	0.5946
CaHK13	MtHK4	0.1962	0.9716
CaHK14	MtHK16	0.0846	0.3489
CaHK15	MtHK7	0.1271	0.3518
CaHK16	MtHK6	0.0337	0.2816
CaHK17	MtHK8	0.1036	0.2919
CaHK18	MtHK20	0.1161	0.7155
CaHK18	MtHK12	0.0663	0.2997
CaHK19	MtHK21	0.0512	0.3815
CaHK3	MtHK19	0.1528	0.7504
CaHK3	MtHK15	0.0513	0.3288
CaHK4	MtHK13	0.1458	0.6476
CaHK4	MtHK1	0.0471	0.302
CaHK5	MtHK3	0.1616	0.8991
CaHK5	MtHK4	0.0271	0.2999
CaHK6	MtHK18	0.1864	0.6622
CaHK6	MtHK5	0.05	0.3627
CaHK7	MtHK20	0.0464	0.2597
CaHK7	MtHK12	0.1103	0.7136
CaHK8	MtHK13	0.0474	0.2866
CaHK8	MtHK1	0.1096	0.6268
CaHK9	MtHK10	0.1178	0.4576
CaHP1	Mt-HP3	0.0407	0.3923
CaHP2	Mt-HP8	0.1982	0.5562
CaHP2	Mt-HP1	0.1036	0.2853
CaHP3	Mt-HP8	0.0476	0.3261
CaHP3	Mt-HP1	0.2313	0.6435
CaHP4	Mt-HP4	0.0316	0.2788
CaHP5	Mt-HP2	0.0604	0.3044
CaHP6	Mt-HP4	0.1257	0.659
CaPHP1	Mt-HP7	0.0931	0.9785
CaPHP1	Mt-HP6	0.0547	0.4435
CaPHP2	Mt-HP7	0.0298	0.3706
CaPHP2	Mt-HP6	0.0848	1.0269
CaPRR1	MtRR56	0.6798	1.6104
CaPRR1	MtRR17	0.0729	0.2836
CaPRR2	MtRR11	0.0987	0.2754
CaPRR3	MtRR56	0.4555	0.9735
CaPRR3	MtRR54	0.119	0.2989
CaPRR4	MtRR33	0.0684	0.2386
CaPRR5	MtRR29	0.12	0.2131
CaRR1	MtRR49	0.0606	0.3452
CaRR12	MtRR32	0.0751	0.3458
CaRR12	MtRR14	0.2081	1.034
CaRR13	MtRR21	0.0786	0.3417
CaRR13	MtRR2	0.323	1.0979
CaRR14	MtRR34	0.204	0.9633
CaRR14	MtRR7	0.1457	0.4259
CaRR15	MtRR21	0.2877	0.8302
CaRR15	MtRR2	0.1917	0.5269
CaRR16	MtRR4	0.0653	0.2337
CaRR17	MtRR60	0.2286	0.6218
CaRR17	MtRR15	0.0694	0.2478
CaRR18	MtRR34	0.0613	0.4243
CaRR18	MtRR7	0.1801	0.8911
CaRR19	MtRR35	0.1742	0.3282
CaRR20	MtRR57	0.3489	0.5051
CaRR21	MtRR39	0.0861	0.3108
CaRR21	MtRR31	0.1742	0.5825
CaRR24	MtRR22	0.6315	0.9074
CaRR25	MtRR39	0.1743	0.6787
CaRR25	MtRR31	0.0847	0.3232
CaRR3	MtRR32	0.1794	0.7151
CaRR3	MtRR14	0.0408	0.4315
CaRR31	MtRR50	0.5928	1.0488
CaRR32	MtRR52	0.0788	0.367
CaRR4	MtRR16	0.1273	0.4429
CaRR5	MtRR40	0.1042	0.3634
CaRR6	MtRR58	0.0536	0.2876
CaRR9	MtRR18	0.0804	0.5836

Ca_TCS	Cc_TCS	Ka	Ks	Ka/Ks
CaHK10	CcHk3	0.1185	0.6293	0.188304465
CaHK12	CcHk18	0.0299	0.518	0.057722008
CaHK13	CcHk6	0.1772	1.0273	0.172490996
CaHK15	CcHk19	0.2484	0.625	0.39744
CaHK4	CcHk14	0.0702	0.4843	0.144951476
CaHK4	CcHk10	0.0916	0.5779	0.158504932
CaHK5	CcHk6	0.0637	0.473	0.134672304
CaHK6	CcRR32	0.1664	0.7821	0.212760517
CaHK6	CcHk20	0.1127	0.5702	0.197649947
CaHK8	CcHk10	0.0716	0.4734	0.151246303
CaPRR1	CcRR34	0.5459	1.8178	0.300308065
CaPRR3	CcRR34	0.2375	0.6138	0.386933855
CaPRR5	CcRR20	0.1624	0.3537	0.459146169
CaRR1	CcRR10	0.0583	0.5124	0.113778298
CaRR12	CcRR6	0.2098	0.6772	0.30980508
CaRR12	CcRR37	0.1801	0.8035	0.224144368
CaRR21	CcRR9	0.1012	0.384	0.263541667
CaRR25	CcRR9	0.1564	0.6856	0.228121354
CaRR3	CcRR37	0.1192	0.5881	0.202686618
CaRR31	CcRR16	0.501	0.6831	0.733421168
CaRR31	CcRR31	0.2881	0.68	0.423676471
CaRR32	CcRR31	0.2007	0.4712	0.425933786
CaRR5	CcRR8	0.1237	0.7046	0.175560602
CaRR9	CcRR3	0.0696	0.3723	0.186946011

Ca_TCS	Gm_TCS	Ka	Ks	Ka/Ks
CaHK1	Glyma.20G090000	0.1234	0.6724	0.183521713
CaHK1	Glyma.19G224200	0.1147	0.6956	0.164893617
CaHK1	Glyma.10G141400	0.1284	0.7263	0.176786452
CaHK1	Glyma.03G227300	0.1768	0.7748	0.228187919
CaHK10	Glyma.16G125100	0.0853	0.3172	0.268915511
CaHK10	Glyma.11G078300	0.1254	0.4135	0.303264813
CaHK10	Glyma.02G046600	0.0835	0.4643	0.17984062
CaHK10	Glyma.01G164800	0.1263	0.4692	0.269181586
CaHK11	Glyma.15G140000	0.0572	1.0535	0.054295206
CaHK11	Glyma.09G035500	0.0585	1.7255	0.033903216
CaHK12	Glyma.19G213300	0.0335	0.6977	0.048014906
CaHK12	Glyma.03G216700	0.0371	0.7828	0.04739397
CaHK13	Glyma.20G202200	0.187	0.5843	0.320041075
CaHK13	Glyma.20G087000	0.3394	0.5843	0.580865993
CaHK13	Glyma.10G188500	0.1914	0.6086	0.314492277
CaHK14	Glyma.14G007100	0.1049	0.8027	0.130683942
CaHK14	Glyma.02G305900	0.1072	0.8168	0.131243879
CaHK15	Glyma.03G219100	0.293	0.643	0.455676516
CaHK16	Glyma.20G090000	0.056	0.582	0.096219931
CaHK16	Glyma.19G224200	0.1038	0.5892	0.176171079
CaHK16	Glyma.10G141400	0.0599	0.6105	0.098116298
CaHK16	Glyma.03G227300	0.1592	0.6444	0.247051521
CaHK18	Glyma.08G105000	0.0911	0.5141	0.177202879
CaHK18	Glyma.05G148100	0.0923	0.5388	0.171306607
CaHK19	Glyma.08G049000	0.0734	0.4741	0.154819658
CaHK19	Glyma.07G173700	0.085	0.4751	0.178909703
CaHK19	Glyma.05G241600	0.0778	0.482	0.161410788
CaHK19	Glyma.02G087000	0.0868	0.4849	0.179005981
CaHK3	Glyma.16G125100	0.1258	0	
CaHK3	Glyma.11G078300	0.0779	0	
CaHK3	Glyma.02G046600	0.1231	0	
CaHK3	Glyma.01G164800	0.0813	0	
CaHK4	Glyma.17G220800	0.082	0.5961	0.137560812
CaHK4	Glyma.14G105400	0.0826	0.6048	0.136574074
CaHK4	Glyma.06G059100	0.1366	0.6396	0.213570982
CaHK4	Glyma.04G058400	0.1287	0.6411	0.200748713
CaHK5	Glyma.20G087000	0.1124	0.5826	0.192928253
CaHK5	Glyma.20G202200	0.0647	0.5878	0.110071453
CaHK5	Glyma.10G188500	0.0608	0.6242	0.097404678
CaHK6	Glyma.20G221800	0.1022	0.5876	0.173927842
CaHK6	Glyma.19G249100	0.1787	0.5942	0.300740491
CaHK6	Glyma.10G167300	0.0978	0.6187	0.15807338
CaHK6	Glyma.03G251700	0.1597	0.6495	0.245881447
CaHK7	Glyma.08G105000	0.0683	0.474	0.144092827
CaHK7	Glyma.05G148100	0.074	0.4817	0.153622587
CaHK8	Glyma.17G220800	0.0898	0.5007	0.179348912
CaHK8	Glyma.14G105400	0.0876	0.5084	0.172305271
CaHK8	Glyma.06G059100	0.0779	0.5226	0.14906238
CaHK8	Glyma.04G058400	0.0673	0.5566	0.120912684
CaHK9	Glyma.15G196500	0.119	0.8643	0.137683675
CaHK9	Glyma.09G088500	0.1149	1.5625	0.073536
CaHP1	Glyma.15G099800	0.0783	1.1071	0.070725318
CaHP1	Glyma.13G212800	0.0783	1.1681	0.067031932
CaHP1	Glyma.10G023200	0.319	1.4637	0.217940835
CaHP1	Glyma.07G253100	0.0907	2.4279	0.037357387
CaHP1	Glyma.02G150800	0.3222	3.2734	0.098429767
CaHP2	Glyma.20G230800	0.1043	0.5863	0.177895275
CaHP2	Glyma.19G239100	0.162	0.5932	0.273095078
CaHP2	Glyma.10G157800	0.0923	0.6161	0.149813342
CaHP2	Glyma.07G253100	0.222	0.631	0.351822504
CaHP2	Glyma.03G241700	0.1527	0.6473	0.235902982
CaHP3	Glyma.20G230800	0.1476	0.665	0.221954887
CaHP3	Glyma.19G239100	0.1175	0.6902	0.17024051
CaHP3	Glyma.10G157800	0.134	0.7251	0.184802096
CaHP3	Glyma.07G253100	0.2837	0.7419	0.382396549
CaHP3	Glyma.03G241700	0.088	0.7592	0.115911486
CaHP4	Glyma.15G019500	0.085	0.9837	0.086408458
CaHP4	Glyma.13G354700	0.0754	1.1261	0.066956753
CaHP4	Glyma.08G201100	0.1289	1.7342	0.074328221
CaHP4	Glyma.07G015600	0.0717	1.8501	0.038754662
CaHP5	Glyma.19G151900	0.1315	0.5946	0.22115708
CaHP5	Glyma.15G099800	0.2867	0.6039	0.474747475
CaHP5	Glyma.13G212800	0.2908	0.6055	0.480264244
CaHP5	Glyma.10G023200	0.0992	0.6287	0.157785907
CaHP5	Glyma.07G253100	0.3084	0.638	0.48338558
CaHP5	Glyma.02G150800	0.1108	0.6508	0.170251998
CaHP6	Glyma.15G019500	0.134	0.4045	0.331273177
CaHP6	Glyma.13G354700	0.1489	0.4081	0.364861554
CaHP6	Glyma.08G201100	0.1072	0.4297	0.249476379
CaHP6	Glyma.07G015600	0.0569	0.4325	0.131560694
CaHP6	Glyma.06G088100	0.2903	0.4387	0.661727832
CaPHP1	Glyma.15G007400	0.0808	1.0004	0.080767693
CaPHP1	Glyma.08G212800	0.0779	1.7411	0.04474183
CaPHP1	Glyma.07G030100	0.0796	2.3137	0.034403769
CaPHP2	Glyma.15G007400	0.0542	0.4712	0.115025467
CaPHP2	Glyma.08G212800	0.0346	0.4743	0.07294961
CaPHP2	Glyma.07G030100	0.0392	0.4808	0.081530782
CaPRR1	Glyma.19G260400	0.5443	0.4929	1.104280787
CaPRR1	Glyma.16G018000	0.507	0.5032	1.007551669
CaPRR1	Glyma.07G049400	0.4952	0.5203	0.951758601
CaPRR1	Glyma.06G136600	0.1459	0.5339	0.273272148
CaPRR1	Glyma.04G228300	0.1364	0.5525	0.246877828
CaPRR1	Glyma.03G261300	0.5514	0.5665	0.973345102
CaPRR2	Glyma.06G196200	0.1099	0.5278	0.208222812
CaPRR2	Glyma.04G166300	0.1101	0.5445	0.202203857
CaPRR3	Glyma.19G260400	0.2163	0.6733	0.321253527
CaPRR3	Glyma.16G018000	0.2997	0.7243	0.413778821
CaPRR3	Glyma.07G049400	0.2693	0.7367	0.36554907
CaPRR3	Glyma.06G136600	0.5304	0.7474	0.709660155
CaPRR3	Glyma.04G228300	0.5268	0.7508	0.701651572
CaPRR3	Glyma.03G261300	0.1969	0.7539	0.261175222
CaPRR4	Glyma.17G102200	0.0956	0	
CaPRR4	Glyma.06G196200	0.1366	0.4384	0.311587591
CaPRR4	Glyma.05G025000	0.0902	0.4461	0.202196817
CaPRR4	Glyma.04G166300	0.1355	0.4602	0.294437201
CaPRR5	Glyma.U034500	0.1581	0.4699	0.336454565
CaPRR5	Glyma.12G073900	0.1406	0.4724	0.297629128
CaRR1	Glyma.19G132300	0.0727	0.7171	0.101380561
CaRR1	Glyma.03G130000	0.0664	0.7961	0.083406607
CaRR11	Glyma.10G269600	0.755	0.6289	1.200508825
CaRR11	Glyma.08G046700	1.0283	0.6296	1.633259212
CaRR12	Glyma.18G130900	0.4221	0	
CaRR12	Glyma.17G093900	0.1537	0	
CaRR12	Glyma.08G292400	0.4097	0.4252	0.963546566
CaRR12	Glyma.06G187000	0.1819	0.4379	0.415391642
CaRR12	Glyma.05G033000	0.1512	0.4448	0.339928058

Mean of Ka and Ks values

Mean Ca_Mt Ka HKs	0.094540741
Mean Ca_Mt Ka RRs	0.186797222
Mean Ca_Cc Ka HKs	0.11502
Mean Ca_Cc Ka RRs	0.210992857
Mean Ca_Gm Ka HKs	0.109243137
Mean Ca_Gm Ka RRs	0.2186
Mean Ca_Mt Ks HKs	0.48737037
Mean Ca_Mt Ks RRs	0.558302778
Mean Ca_Cc Ks HKs	0.61605
Mean Ca_Cc Ks RRs	0.667664286
Mean Ca_Gm Ks HKs	0.596560784
Mean Ca_Gm Ks RRs	0.528914118

Ratio of Ka and Ks values RRs Vs HKs

Ca_Mt	
Ka_RRs/Ka_HKs	1.975838361
Ca_Cc	
Ka_RRs/Ka_HKs	1.834401471
Ca_Gm	
Ka_RRs/Ka_HKs	2.001041031
Ca_Mt	
Ks_RRs/Ks_HKs	1.145541075
Ca_Cc	
Ks_RRs/Ks_HKs	1.083782624
Ca_Gm	
Ks_RRs/Ks_HKs	0.886605576

CaRR12	Glyma.04G177900	0.1785	0.4579	0.389823105
CaRR12	Glyma.02G027400	0.3698	0.4674	0.79118528
CaRR12	Glyma.01G037500	0.3622	0.4685	0.773105656
CaRR13	Glyma.17G217100	0.2252	0.5029	0.447802744
CaRR13	Glyma.14G110600	0.2326	0.5036	0.461874504
CaRR13	Glyma.06G063500	0.1597	0.5231	0.305295355
CaRR13	Glyma.04G062500	0.1558	0.5601	0.278164613
CaRR14	Glyma.17G030600	0.1755	0.8558	0.205071278
CaRR14	Glyma.15G145200	0.1647	0.9407	0.175082385
CaRR14	Glyma.09G040000	0.1699	1.7179	0.09889982
CaRR14	Glyma.07G243300	0.1717	2.0549	0.083556377
CaRR15	Glyma.17G217100	0.2077	0.6037	0.344045055
CaRR15	Glyma.14G110600	0.2054	0.6046	0.339728746
CaRR15	Glyma.06G063500	0.2836	0.6405	0.442779079
CaRR15	Glyma.04G062500	0.2481	0.6415	0.386749805
CaRR16	Glyma.07G171200	0.1543	0.6374	0.242077189
CaRR16	Glyma.02G085900	0.1421	0.65	0.218615385
CaRR17	Glyma.18G010800	0.1301	0.4977	0.261402451
CaRR17	Glyma.11G246400	0.1223	0.5119	0.23891385
CaRR17	Glyma.08G100900	0.1853	0.5124	0.361631538
CaRR17	Glyma.05G144500	0.1698	0.5356	0.317027633
CaRR18	Glyma.17G030600	0.0817	0	
CaRR18	Glyma.15G145200	0.1557	0.3842	0.405257678
CaRR18	Glyma.09G040000	0.1561	0.4215	0.370344009
CaRR18	Glyma.07G243300	0.0784	0.4367	0.17952828
CaRR19	Glyma.17G076000	0.2287	0	
CaRR19	Glyma.13G155400	0.25	0.4047	0.617741537
CaRR21	Glyma.17G152800	0.1581	0.5334	0.29640045
CaRR21	Glyma.11G041300	0.101	0	
CaRR21	Glyma.05G070200	0.1599	0	
CaRR21	Glyma.01G200800	0.1061	0	
CaRR25	Glyma.17G152800	0.1401	0.3075	0.455609756
CaRR25	Glyma.11G041300	0.1584	0.4153	0.381411028
CaRR25	Glyma.05G070200	0.1292	0.441	0.292970522
CaRR25	Glyma.01G200800	0.157	0.4697	0.334255908
CaRR3	Glyma.18G130900	0.425	0.4962	0.856509472
CaRR3	Glyma.17G093900	0.1819	0.5018	0.362495018
CaRR3	Glyma.08G292400	0.4023	0.5196	0.774249423
CaRR3	Glyma.06G187000	0.0993	0.5308	0.187076112
CaRR3	Glyma.05G033000	0.1813	0.5401	0.335678578
CaRR3	Glyma.04G177900	0.0953	0.5487	0.173683251
CaRR3	Glyma.02G027400	0.3101	0.5725	0.541659389
CaRR3	Glyma.01G037500	0.3371	0.5766	0.584634062
CaRR31	Glyma.19G169700	0.2857	0.7022	0.406864141
CaRR32	Glyma.19G171300	0.1952	0.6999	0.278896985
CaRR32	Glyma.03G170100	0.1382	0.7905	0.174826059
CaRR4	Glyma.06G114900	0.1332	0.5345	0.249204864
CaRR4	Glyma.04G247800	0.1305	0.5526	0.236156352
CaRR5	Glyma.02G027400	0.1449	0.488	0.29692623
CaRR5	Glyma.01G037500	0.1096	0.4901	0.223627831
CaRR6	Glyma.15G236200	0.0684	0.3526	0.193987521
CaRR6	Glyma.13G197600	0.0724	0.4108	0.17624148
CaRR6	Glyma.11G155100	0.1151	0.4129	0.27875999
CaRR6	Glyma.04G137600	0.1357	0.4556	0.29784899
CaRR9	Glyma.06G142300	0.0506	0.5329	0.094952149
CaRR9	Glyma.04G223000	0.0573	0.5521	0.103785546

Supplemental Table 3C: A list of orthologs pairs in *Medicago -Glycine, Medicago-Cajanus* and *Cajanus-Glycine*

Mt_TCS	Gm_TCS	Ka	Ks
MtHK1	Glyma.17G220800	0.0801	0.5022
MtHK1	Glyma.14G105400	0.0848	0.5031
MtHK1	Glyma.06G059100	0.1333	0.6808
MtHK1	Glyma.04G058400	0.1193	0.7253
MtHK10	Glyma.15G196500	0.1249	0.4399
MtHK10	Glyma.09G088500	0.1166	0.4336
MtHK12	Glyma.08G105000	0.0967	0.6159
MtHK12	Glyma.05G148100	0.1027	0.642
MtHK13	Glyma.17G220800	0.1269	0.6405
MtHK13	Glyma.14G105400	0.1293	0.6713
MtHK13	Glyma.06G059100	0.1065	0.5443
MtHK13	Glyma.04G058400	0.1128	0.616
MtHK14	Glyma.09G002600	0.0544	0.394
MtHK15	Glyma.16G125100	0.1237	0.7055
MtHK15	Glyma.11G078300	0.0785	0.6009
MtHK15	Glyma.02G046600	0.1263	0.6923
MtHK15	Glyma.01G164800	0.0822	0.5878
MtHK16	Glyma.14G007100	0.1196	0.4618
MtHK16	Glyma.02G305900	0.1219	0.4674
MtHK17	Glyma.19G213300	0.0346	0.549
MtHK17	Glyma.03G216700	0.0365	0.5367
MtHK18	Glyma.20G221800	0.1493	0.6807
MtHK18	Glyma.19G249100	0.1167	0.5173
MtHK18	Glyma.10G167300	0.1589	0.7265
MtHK18	Glyma.03G251700	0.0975	0.5076
MtHK19	Glyma.16G125100	0.0948	0.459
MtHK19	Glyma.11G078300	0.1314	0.6034
MtHK19	Glyma.02G046600	0.0957	0.4328
MtHK19	Glyma.01G164800	0.1308	0.6424
MtHK20	Glyma.08G105000	0.0739	0.4819
MtHK20	Glyma.05G148100	0.0775	0.4641
MtHK21	Glyma.08G049000	0.0741	0.5304
MtHK21	Glyma.07G173700	0.0918	0.6537
MtHK21	Glyma.05G241600	0.0792	0.5204
MtHK21	Glyma.02G087000	0.089	0.6262
MtHK3	Glyma.20G202200	0.1749	0.7393
MtHK3	Glyma.20G087000	0.1579	0.7409
MtHK3	Glyma.10G188500	0.1757	0.7372
MtHK3	Glyma.10G008500	0.339	0.9823
MtHK4	Glyma.20G087000	0.1219	0.8304
MtHK4	Glyma.20G202200	0.0745	0.4654
MtHK4	Glyma.10G008500	0.2768	0.7757
MtHK4	Glyma.10G188500	0.0696	0.4771
MtHK5	Glyma.20G221800	0.1089	0.6569
MtHK5	Glyma.19G249100	0.1801	0.8039
MtHK5	Glyma.10G167300	0.1059	0.6664
MtHK5	Glyma.03G251700	0.1582	0.7926
MtHK6	Glyma.20G090000	0.0649	0.5142
MtHK6	Glyma.19G224200	0.1081	0.6795
MtHK6	Glyma.10G141400	0.0925	0.5497
MtHK6	Glyma.03G227300	0.1712	0.7508
MtHK7	Glyma.03G219100	0.3146	0.8005
MtHK9	Glyma.15G140000	0.0732	0.6866
MtHK9	Glyma.09G035500	0.0708	0.6788
Mt-HP1	Glyma.20G230800	0.1526	0.4655
Mt-HP1	Glyma.19G239100	0.2229	0.488
Mt-HP1	Glyma.10G157800	0.1358	0.4096
Mt-HP1	Glyma.03G241700	0.1858	0.5869
Mt-HP2	Glyma.19G151900	0.1691	0.5089
Mt-HP2	Glyma.13G212800	0.3224	0
Mt-HP2	Glyma.10G023200	0.1088	0.6736
Mt-HP2	Glyma.07G253100	0.3333	0
Mt-HP2	Glyma.03G148600	0.1912	0.6158
Mt-HP2	Glyma.02G150800	0.1273	0.6806
Mt-HP3	Glyma.15G099800	0.1081	0.5146
Mt-HP3	Glyma.13G212800	0.0896	0.4571
Mt-HP3	Glyma.07G253100	0.1058	0.6411
Mt-HP3	Glyma.02G150800	0.322	0
Mt-HP4	Glyma.15G019500	0.0774	0.4765
Mt-HP4	Glyma.13G354700	0.0741	0.4071
Mt-HP4	Glyma.08G201100	0.1192	0.8399
Mt-HP4	Glyma.07G015600	0.0639	0.7289
Mt-HP6	Glyma.15G007400	0.0664	0.4517
Mt-HP6	Glyma.08G212800	0.0521	0.7439
Mt-HP6	Glyma.07G030100	0.0537	0.6266
Mt-HP7	Glyma.15G007400	0.0481	0.7925
Mt-HP7	Glyma.08G212800	0.0316	0.7989
Mt-HP7	Glyma.07G030100	0.0363	0.7455
Mt-HP8	Glyma.20G230800	0.1653	0.8319
Mt-HP8	Glyma.19G239100	0.1134	0.5605
Mt-HP8	Glyma.10G157800	0.1381	0.7005
Mt-HP8	Glyma.03G241700	0.0874	0.538
MtRR11	Glyma.17G102200	0.1615	0.4736
MtRR11	Glyma.06G196200	0.1211	0.4257
MtRR11	Glyma.04G166300	0.1183	0.3872
MtRR15	Glyma.18G010700	0.1177	0.3487
MtRR15	Glyma.11G246400	0.108	0.4984
MtRR15	Glyma.08G100900	0.1805	0.6107
MtRR15	Glyma.05G144500	0.1632	0.5575
MtRR17	Glyma.19G260400	0.5833	1.4378
MtRR17	Glyma.16G018000	0.6363	2.0451
MtRR17	Glyma.07G049400	0.5278	1.7493
MtRR17	Glyma.06G136600	0.1283	0.4896
MtRR17	Glyma.04G228300	0.1257	0.4481
MtRR17	Glyma.03G261300	0.5955	1.5717
MtRR29	Glyma.U034500	0.1647	0.3231
MtRR29	Glyma.12G073900	0.1446	0.3268
MtRR31	Glyma.17G152800	0.1487	0.4163
MtRR31	Glyma.11G041300	0.1612	0.5606
MtRR31	Glyma.05G070200	0.1379	0.3994
MtRR31	Glyma.01G200800	0.1617	0.5226
MtRR33	Glyma.17G102200	0.1158	0.469
MtRR33	Glyma.06G196200	0.1486	0.5364
MtRR33	Glyma.05G025000	0.1062	0.4361
MtRR33	Glyma.04G166300	0.1575	0.4895
MtRR34	Glyma.17G030600	0.0954	0.6505
MtRR34	Glyma.15G145200	0.1523	0.8091
MtRR34	Glyma.09G040000	0.1613	0.8218
MtRR34	Glyma.07G243300	0.093	0.6241
MtRR39	Glyma.17G152800	0.1684	0.5349
MtRR39	Glyma.11G041300	0.1133	0.4906
MtRR39	Glyma.05G070200	0.1676	0.4908
MtRR39	Glyma.01G200800	0.118	0.4689
MtRR4	Glyma.08G046800	0.523	0.7956
MtRR4	Glyma.07G171200	0.1756	0.4237
MtRR4	Glyma.02G085900	0.1608	0.3867
MtRR50	Glyma.19G169700	0.5449	0.941
MtRR52	Glyma.19G171300	0.1768	0.6074
MtRR52	Glyma.03G170100	0.1357	0.4539
MtRR54	Glyma.19G260400	0.2129	0.5374
MtRR54	Glyma.16G018000	0.2994	0.6243
MtRR54	Glyma.07G049400	0.2979	0.6122
MtRR54	Glyma.06G136600	0.5405	1.786
MtRR54	Glyma.04G228300	0.5449	1.5964
MtRR54	Glyma.03G261300	0.2163	0.5291
MtRR56	Glyma.19G260400	0.36	0.6598
MtRR56	Glyma.16G018000	0.3392	0.6241
MtRR56	Glyma.07G049400	0.3139	0.586
MtRR56	Glyma.06G136600	0.6872	2.0726
MtRR56	Glyma.04G228300	0.6559	2.03
MtRR56	Glyma.03G261300	0.3704	0.6954
MtRR57	Glyma.07G079000	0.5143	0.6344
MtRR57	Glyma.03G017700	0.8559	1.4142
MtRR6	Glyma.13G135900	0.1027	0.3706
MtRR6	Glyma.12G073900	0.4732	1.5787
MtRR6	Glyma.10G048100	0.11	0.3275
MtRR60	Glyma.18G010700	0.2508	0.7562

Mt_TCS	Cc_TCS	Ka	Ks	Ka/Ks
MtHK1	CcHk14	0.0921	0.504	0.182738095
MtHK13	CcHk10	0.0962	0.5178	0.185786018
MtHK15	CcHk3	0.0841	0.5958	0.14115475
MtHK19	CcHk3	0.1344	0.6997	0.192082321
MtHK6	CcHk11	0.0867	0.7623	0.11373475
MtHK7	CcHk19	0.2956	0.6369	0.464123096
MtHK9	CcHk12	0.0847	0.9856	0.0859375
MtRR11	CcRR29	0.1185	0.403	0.294044665
MtRR17	CcRR34	0.5995	1.5973	0.375320854
MtRR29	CcRR20	0.1704	0.3584	0.475446429
MtRR31	CcRR9	0.1655	0.6335	0.26124704
MtRR39	CcRR9	0.1211	0.475	0.254947368
MtRR51	CcRR16	0.4927	0.7912	0.622724975
MtRR52	CcRR31	0.195	0.5348	0.364622289
MtRR54	CcRR34	0.212	0.5612	0.377761939
MtRR56	CcRR34	0.4733	0.89	0.531797753
MtRR57	CcRR23	0.5066	0.5621	0.90126312
MtRR6	CcRR20	0.4728	1.8845	0.25088883

Cc_TCS	Gm_TCS	Ka	Ks	Ka/Ks
CcHk1	Glyma.08G105000	0.0324	0.1781	0.18192027
CcHk1	Glyma.05G148100	0.0348	0.1708	0.203747073
CcHk10	Glyma.06G059100	0.0346	0.1841	0.187941336
CcHk10	Glyma.04G058400	0.0417	0.2177	0.191548002
CcHk11	Glyma.20G090000	0.0849	0.6086	0.139500493
CcHk11	Glyma.19G224200	0.0491	0.2588	0.189721793
CcHk11	Glyma.10G141400	0.0919	0.5884	0.156186268
CcHk11	Glyma.03G227300	0.1124	0.3199	0.3513598
CcHk12	Glyma.15G140000	0.0522	0.5075	0.102857143
CcHk12	Glyma.09G035500	0.0504	0.5231	0.09634869
CcHk13	Glyma.14G007100	0.0655	0.2181	0.300320954
CcHk13	Glyma.02G305900	0.0643	0.198	0.324747475
CcHk14	Glyma.17G220800	0.0303	0.1854	0.163430421
CcHk14	Glyma.14G105400	0.0303	0.2006	0.151046859
CcHk14	Glyma.06G059100	0.1031	0.4799	0.214836424
CcHk14	Glyma.04G058400	0.1156	0.5582	0.207094231
CcHk18	Glyma.19G213300	0.0118	0.3145	0.037519873
CcHk18	Glyma.03G216700	0.0132	0.3009	0.043868395
CcHk3	Glyma.16G125100	0.1096	0.4818	0.227480282
CcHk3	Glyma.11G078300	0.0356	0.2222	0.160216022
CcHk3	Glyma.02G046600	0.1068	0.4937	0.216325704
CcHk3	Glyma.01G164800	0.0379	0.2065	0.183535109
CcHk5	Glyma.07G173700	0.0316	0.2559	0.123485737
CcHk5	Glyma.02G087000	0.0301	0.23	0.130869565
CcHk6	Glyma.10G008500	0.2513	0.6994	0.359307978
CcHk7	Glyma.08G049000	0.0473	0.2271	0.208278292
CcHk7	Glyma.07G173700	0.0862	0.4924	0.175060926
CcHk7	Glyma.05G241600	0.0485	0.2169	0.223605348
CcHk7	Glyma.02G087000	0.0865	0.5146	0.168091722
CcHP1	Glyma.19G239100	0.1208	0.2981	0.405233143
CcHP1	Glyma.03G241700	0.0862	0.3234	0.266542981
CcHP10	Glyma.06G088100	0.1431	0.3628	0.394432194
CcHP2	Glyma.19G151900	0.2098	0.5278	0.397499053
CcHP2	Glyma.15G099800	0.3105	1.9595	0.158458791
CcHP2	Glyma.13G212800	0.3229	1.9792	0.163146726
CcHP2	Glyma.10G023200	0.0831	0.2426	0.342539159
CcHP2	Glyma.02G150800	0.0738	0.2441	0.302335109
CcHP7	Glyma.15G099800	0.0693	0.4649	0.149064315
CcHP7	Glyma.13G212800	0.0707	0.4956	0.142655367
CcHP7	Glyma.07G253100	0.0271	0.2002	0.135364635
CcHP8	Glyma.19G151900	0.2698	0	
CcHP8	Glyma.15G099800	0.037	0.1961	0.188679245
CcHP8	Glyma.13G212800	0.0369	0.2656	0.138930723
CcHP8	Glyma.07G253100	0.1029	0.65	0.158307692
CcHP8	Glyma.02G150800	0.3043	2.7807	0.109432877
CcHP9	Glyma.20G230800	0.0618	0.2613	0.236509759
CcHP9	Glyma.19G239100	0.1684	0.5949	0.283072785
CcHP9	Glyma.10G157800	0.0652	0.2295	0.284095861
CcHP9	Glyma.03G241700	0.1736	0.7	0.248
CcRR1	Glyma.18G010800	0.1558	0.4721	0.330014827
CcRR1	Glyma.11G246400	0.1514	0.5192	0.291602465
CcRR1	Glyma.08G100900	0.0547	0.1982	0.275983855
CcRR1	Glyma.05G144500	0.0419	0.1845	0.227100271
CcRR10	Glyma.19G132300	0.0412	0.1715	0.240233236
CcRR10	Glyma.03G130000	0.0634	0.1981	0.320040384
CcRR15	Glyma.08G046700	0.5513	0.7906	0.697318492
CcRR16	Glyma.19G169700	0.4205	0.426	0.987089202
CcRR18	Glyma.13G077500	0.0745	0.2411	0.309000415
CcRR18	Glyma.06G114900	0.2402	0.6273	0.382910888
CcRR18	Glyma.04G247800	0.2294	0.7092	0.323463057
CcRR20	Glyma.U034500	0.0881	0.1832	0.480895197
CcRR20	Glyma.13G135900	0.4536	1.4452	0.313866593
CcRR20	Glyma.12G073900	0.0605	0.1546	0.391332471
CcRR20	Glyma.10G048100	0.4571	1.5102	0.302675142
CcRR21	Glyma.08G100900	0.0802	0.402	0.199502488
CcRR21	Glyma.05G144500	0.0809	0.4203	0.192481561
CcRR23	Glyma.07G079000	0.2601	0.3692	0.704496208
CcRR23	Glyma.03G017700	0.3231	0.4196	0.770019066
CcRR24	Glyma.09G040000	0.0772	0.2548	0.302982732
CcRR29	Glyma.06G196200	0.0465	0.2035	0.228501229
CcRR3	Glyma.06G142300	0.0161	0.1403	0.114754098
CcRR30	Glyma.17G217100	0.1918	0.4825	0.397512953
CcRR30	Glyma.14G110600	0.1982	0.5227	0.379185001
CcRR30	Glyma.06G063500	0.0859	0.2431	0.35335253
CcRR30	Glyma.04G062500	0.0687	0.2165	0.317321016
CcRR31	Glyma.19G171300	0.2377	0.4679	0.508014533
CcRR31	Glyma.03G170100	0.1203	0.2233	0.538737125
CcRR33	Glyma.19G250500	0.0929	0.3065	0.303099511
CcRR33	Glyma.03G252900	0.2344	0.4673	0.501604965
CcRR34	Glyma.19G260400	0.0633	0.2075	0.305060241
CcRR34	Glyma.16G018000	0.2713	0.585	0.463760684
CcRR34	Glyma.07G049400	0.2476	0.5737	0.431584452
CcRR34	Glyma.06G136600	0.5561	2.1401	0.259847671
CcRR34	Glyma.04G228300	0.5244	1.9736	0.265707337
CcRR34	Glyma.03G261300	0.0704	0.2068	0.340425532
CcRR37	Glyma.06G187000	0.0708	0.3561	0.198820556
CcRR37	Glyma.05G033000	0.1843	0.8178	0.225360724
CcRR37	Glyma.04G177900	0.0803	0.2946	0.27257298
CcRR6	Glyma.17G093900	0.1227	0.3553	0.345341965
CcRR6	Glyma.06G187000	0.2235	0.8508	0.262693935
CcRR6	Glyma.05G033000	0.1138	0.3532	0.322197055
CcRR8	Glyma.18G130900	0.1667	0.8243	0.202232197
CcRR8	Glyma.08G292400	0.2103	0.7224	0.291112957
CcRR8	Glyma.02G027400	0.0519	0.4069	0.127549767
CcRR8	Glyma.01G037500	0.0695	0.3968	0.17515121
CcRR9	Glyma.17G152800	0.1485	0.5518	0.269119246
CcRR9	Glyma.11G041300	0.0437	0.1933	0.226073461
CcRR9	Glyma.05G070200	0.146	0.5339	0.273459449
CcRR9	Glyma.01G200800	0.0478	0.176	0.271590909

Mean of Ka and Ks values

Mean Mt_Gm Ka HKs	0.11872
Mean Mt_Gm Ka RRs	0.259294
Mean Mt_Cc Ka HKs	0.124829
Mean Mt_Cc Ka RRs	0.320673
Mean Cc_Gm Ka HKs	0.065169
Mean Cc_Gm Ka RRs	0.17221
Mean Mt_Gm Ks HKs	0.614535
Mean Mt_Gm Ks RRs	0.730618
Mean Mt_Cc Ks HKs	0.671729
Mean Mt_Cc Ks RRs	0.790091
Mean Cc_Gm Ks HKs	0.346659
Mean Cc_Gm Ks RRs	0.509808

Ratio of Ka and Ks values RRs Vs HKs

Mt_Gm	
Ka_RRs/Ka_HKs	2.18407
Mt_Cc	
Ka_RRs/Ka_HKs	2.568905
Cc_Gm	
Ka_RRs/Ka_HKs	2.642515
Mt_Gm	
Ks_RRs/Ks_HKs	1.188895
Mt_Cc	
Ks_RRs/Ks_HKs	1.176206
Cc_Gm	
Ks_RRs/Ks_HKs	1.470634

MtRR60	Glyma.11G246400	0.1947	0.6063
MtRR60	Glyma.08G100900	0.1412	0.4183
MtRR60	Glyma.05G144500	0.1399	0.4161
MtRR7	Glyma.17G030600	0.1815	0.6728
MtRR7	Glyma.15G145200	0.1521	0.5421
MtRR7	Glyma.09G040000	0.1489	0.5045
MtRR7	Glyma.07G243300	0.1723	0.6811

Supplemental Table 4: Pairwise comparison of segmentally duplicated TCS pairs present on chromosome in legumes.

Chickpea-*Medicago*			
Ca_TCS	Chr no.	MtTCS	Chr no.
CaRR14	1	MtRR34	chr4
CaPHP1	1	Mt-HP7	chr4
CaHP4	1	Mt-HP4	chr2
CaPHP1	1	Mt-HP6	chr2
CaHK9	1	MtHK10	chr2
CaRR14	1	MtRR7	chr2
CaHK11	1	MtHK9	chr2
CaHP1	1	Mt-HP3	chr2
CaHK14	2	MtHK16	chr5
CaPRR3	3	MtRR56	chr8
CaPRR3	3	MtRR54	chr7
CaHP3	3	Mt-HP8	chr7
CaRR32	3	MtRR52	chr7
CaRR31	3	MtRR50	chr7
CaRR1	3	MtRR49	chr7
CaHP3	3	Mt-HP1	chr1
CaHK1	3	MtHK6	chr1
CaHP2	4	Mt-HP8	chr7
CaHK6	4	MtHK18	chr7
CaHK4	4	MtHK13	chr3
CaRR15	4	MtRR21	chr3
CaHK5	4	MtHK3	chr1
CaHK13	4	MtHK3	chr1
CaRR16	4	MtRR4	chr1
CaHK13	4	MtHK4	chr1
CaHK4	4	MtHK1	chr1
CaRR15	4	MtRR2	chr1
CaHK17	4	MtHK8	chr2
CaHP5	4	Mt-HP2	chr1
CaHK15	4	MtHK7	chr1
CaHK16	4	MtHK6	chr1
CaHP2	4	Mt-HP1	chr1
CaHK6	4	MtHK5	chr1
CaHK5	4	MtHK4	chr1
CaPRR1	5	MtRR56	chr8
CaRR17	5	MtRR60	chr8
CaHK18	5	MtHK20	chr8
CaRR3	5	MtRR32	chr4
CaPRR2	5	MtRR11	chr3
CaHK8	5	MtHK13	chr3
CaRR13	5	MtRR21	chr3
CaRR9	5	MtRR18	chr3
CaPRR1	5	MtRR17	chr3
CaRR4	5	MtRR16	chr3
CaRR17	5	MtRR15	chr3
CaHK18	5	MtHK12	chr3
CaRR3	5	MtRR14	chr3
CaHK8	5	MtHK1	chr1
CaRR13	5	MtRR2	chr1
CaHK7	6	MtHK20	chr8
CaHK19	6	MtHK21	chr8
CaRR5	6	MtRR40	chr5
CaRR24	6	MtRR22	chr4
CaPHP2	6	Mt-HP7	chr4
CaPRR5	6	MtRR29	chr4
CaHK7	6	MtHK12	chr3
CaPHP2	6	Mt-HP6	chr2
CaHK10	7	MtHK19	chr8
CaRR6	7	MtRR58	chr8
CaRR20	7	MtRR57	chr8
CaHK10	7	MtHK15	chr5
CaRR25	7	MtRR39	chr5
CaRR25	7	MtRR31	chr4
CaRR12	7	MtRR32	chr4
CaPRR4	7	MtRR33	chr4
CaRR18	7	MtRR34	chr4
CaRR19	7	MtRR35	chr4
CaRR12	7	MtRR14	chr3
CaHP6	7	Mt-HP4	chr2
CaRR18	7	MtRR7	chr2
CaRR21	8	MtRR39	chr5
CaRR21	8	MtRR31	chr4
CaHK12	Scaffold943	MtHK17	chr7
CaHK3	8	MtHK15	chr5
CaHK3	8	MtHK19	chr8

Chickpea-Soybean			
Ca_TCS	Chr no.	GmTCS	Chr no.
CaRR14	1	Glyma.17G030600	17
CaHK9	1	Glyma.15G196500	15
CaRR14	1	Glyma.15G145200	15
CaHP4	1	Glyma.15G019500	15
CaPHP1	1	Glyma.15G007400	15
CaHK11	1	Glyma.15G140000	15
CaHP1	1	Glyma.15G099800	15
CaHP4	1	Glyma.13G354700	13
CaHP1	1	Glyma.13G212800	13
CaHP1	1	Glyma.10G023200	10
CaHK9	1	Glyma.09G088500	9
CaRR14	1	Glyma.09G040000	9
CaHK11	1	Glyma.09G035500	9
CaHP4	1	Glyma.08G201100	8
CaPHP1	1	Glyma.08G212800	8
CaHP4	1	Glyma.07G015600	7
CaRR14	1	Glyma.07G243300	7
CaPHP1	1	Glyma.07G030100	7
CaHP1	1	Glyma.07G253100	7
CaHP1	1	Glyma.02G150800	2
CaHK14	2	Glyma.14G007100	14
CaHK14	2	Glyma.02G305900	2
CaHP3	3	Glyma.20G230800	20
CaHK1	3	Glyma.20G090000	20
CaPRR3	3	Glyma.19G260400	19
CaHP3	3	Glyma.19G239100	19
CaHK1	3	Glyma.19G224200	19
CaRR32	3	Glyma.19G171300	19
CaRR31	3	Glyma.19G169700	19
CaRR1	3	Glyma.19G132300	19
CaPRR3	3	Glyma.16G018000	16
CaHP3	3	Glyma.10G157800	10
CaHK1	3	Glyma.10G141400	10
CaPRR3	3	Glyma.07G049400	7
CaHP3	3	Glyma.07G253100	7
CaPRR3	3	Glyma.06G136600	6
CaPRR3	3	Glyma.04G228300	4
CaPRR3	3	Glyma.03G261300	3
CaHP3	3	Glyma.03G241700	3
CaHK1	3	Glyma.03G227300	3
CaRR32	3	Glyma.03G170100	3
CaRR1	3	Glyma.03G130000	3
CaHK16	4	Glyma.20G090000	20
CaHK5	4	Glyma.20G087000	20
CaHK13	4	Glyma.20G202200	20
CaHK13	4	Glyma.20G087000	20
CaHP2	4	Glyma.20G230800	20
CaHK6	4	Glyma.20G221800	20
CaHK5	4	Glyma.20G202200	20
CaHK16	4	Glyma.19G224200	19
CaHP2	4	Glyma.19G239100	19
CaHK6	4	Glyma.19G249100	19
CaHP5	4	Glyma.19G151900	19
CaHK4	4	Glyma.17G220800	17
CaRR15	4	Glyma.17G217100	17
CaHP5	4	Glyma.15G099800	15
CaRR15	4	Glyma.14G110600	14
CaHK4	4	Glyma.14G105400	14
CaHP5	4	Glyma.13G212800	13
CaHK13	4	Glyma.10G188500	10
CaHK16	4	Glyma.10G141400	10
CaHP2	4	Glyma.10G157800	10
CaHK6	4	Glyma.10G167300	10
CaHK5	4	Glyma.10G188500	10
CaHP5	4	Glyma.10G023200	10
CaRR11	4	Glyma.10G269600	10
CaRR11	4	Glyma.08G046700	8
CaHP2	4	Glyma.07G253100	7
CaRR16	4	Glyma.07G171200	7
CaHP5	4	Glyma.07G253100	7
CaHK4	4	Glyma.06G059100	6
CaRR15	4	Glyma.06G063500	6
CaHK4	4	Glyma.04G058400	4
CaRR15	4	Glyma.04G062500	4
CaHK15	4	Glyma.03G219100	3
CaHK16	4	Glyma.03G227300	3
CaHP2	4	Glyma.03G241700	3
CaHK6	4	Glyma.03G251700	3
CaRR16	4	Glyma.02G085900	2
CaHP5	4	Glyma.02G150800	2
CaPRR1	5	Glyma.19G260400	19
CaRR3	5	Glyma.18G130900	18
CaRR17	5	Glyma.18G010800	18
CaHK8	5	Glyma.17G220800	17
CaRR3	5	Glyma.17G093900	17
CaRR13	5	Glyma.17G217100	17
CaPRR1	5	Glyma.16G018000	16
CaRR13	5	Glyma.14G110600	14
CaHK8	5	Glyma.14G105400	14
CaRR17	5	Glyma.11G246400	11
CaRR17	5	Glyma.08G100900	8
CaHK18	5	Glyma.08G105000	8
CaRR3	5	Glyma.08G292400	8
CaPRR1	5	Glyma.07G049400	7
CaHK8	5	Glyma.06G059100	6
CaRR13	5	Glyma.06G063500	6
CaPRR2	5	Glyma.06G196200	6
CaRR3	5	Glyma.06G187000	6
CaRR9	5	Glyma.06G142300	6
CaPRR1	5	Glyma.06G136600	6
CaRR4	5	Glyma.06G114900	6
CaRR17	5	Glyma.05G144500	5
CaHK18	5	Glyma.05G148100	5
CaRR3	5	Glyma.05G033000	5
CaPRR2	5	Glyma.04G166300	4
CaRR3	5	Glyma.04G177900	4
CaRR9	5	Glyma.04G223000	4
CaPRR1	5	Glyma.04G228300	4
CaRR4	5	Glyma.04G247800	4
CaHK8	5	Glyma.04G058400	4
CaRR13	5	Glyma.04G062500	4
CaPRR1	5	Glyma.03G261300	3
CaRR3	5	Glyma.02G027400	2
CaRR3	5	Glyma.01G037500	1
CaPRR5	6	Glyma.U034500	U
CaPHP2	6	Glyma.15G007400	15
CaPRR5	6	Glyma.12G073900	12
CaHK7	6	Glyma.08G105000	8
CaHK19	6	Glyma.08G049000	8
CaPHP2	6	Glyma.08G212800	8
CaHK19	6	Glyma.07G173700	7
CaPHP2	6	Glyma.07G030100	7
CaHK7	6	Glyma.05G148100	5
CaHK19	6	Glyma.05G241600	5
CaHK19	6	Glyma.02G087000	2
CaRR5	6	Glyma.02G027400	2
CaRR5	6	Glyma.01G037500	1
CaRR12	7	Glyma.18G130900	18
CaRR12	7	Glyma.17G093900	17
CaPRR4	7	Glyma.17G102200	17
CaRR18	7	Glyma.17G030600	17
CaRR19	7	Glyma.17G076000	17
CaRR25	7	Glyma.17G152800	17
CaHK10	7	Glyma.16G125100	16
CaRR6	7	Glyma.15G236200	15
CaRR18	7	Glyma.15G145200	15
CaHP6	7	Glyma.15G019500	15
CaRR19	7	Glyma.13G155400	13
CaHP6	7	Glyma.13G354700	13
CaRR6	7	Glyma.13G197600	13
CaRR6	7	Glyma.11G155100	11
CaHK10	7	Glyma.11G078300	11
CaRR25	7	Glyma.11G041300	11
CaRR18	7	Glyma.09G040000	9
CaRR12	7	Glyma.08G292400	8
CaHP6	7	Glyma.08G201100	8
CaHP6	7	Glyma.07G015600	7
CaRR18	7	Glyma.07G243300	7
CaRR12	7	Glyma.06G187000	6
CaPRR4	7	Glyma.06G196200	6
CaHP6	7	Glyma.06G088100	6
CaRR25	7	Glyma.05G070200	5
CaRR12	7	Glyma.05G033000	5
CaPRR4	7	Glyma.05G025000	5
CaRR6	7	Glyma.04G137600	4
CaRR12	7	Glyma.04G177900	4
CaPRR4	7	Glyma.04G166300	4
CaHK10	7	Glyma.02G046600	2
CaRR12	7	Glyma.02G027400	2
CaRR12	7	Glyma.01G037500	1
CaHK10	7	Glyma.01G164800	1
CaRR25	7	Glyma.01G200800	1
CaRR21	8	Glyma.17G152800	17
CaRR21	8	Glyma.11G041300	11
CaRR21	8	Glyma.05G070200	5
CaRR21	8	Glyma.01G200800	1
CaHK12	Scaffold943	Glyma.19G213300	19
CaHK12	Scaffold943	Glyma.03G216700	3
CaHK3	8	Glyma.16G125100	16
CaHK3	8	Glyma.11G078300	11
CaHK3	8	Glyma.02G046600	2
CaHK3	8	Glyma.01G164800	1

Chickpea-*C. cajan*			
CcTCS	Chr no.	Ca_TCS	Chr no.
CcHk19	CcLG02	CaHK15	4
CcHk20	CcLG02	CaHK6	4
CcRR34	CcLG03	CaPRR1	5
CcHk10	CcLG03	CaHK8	5
CcRR32	CcLG03	CaHK6	4
CcHk10	CcLG03	CaHK4	4
CcRR31	CcLG03	CaRR32	3
CcRR34	CcLG03	CaPRR3	3
CcRR31	CcLG03	CaRR31	3
CcRR20	CcLG04	CaPRR5	6
CcHk3	CcLG06	CaHK3	8
CcRR9	CcLG06	CaRR21	8
CcHk3	CcLG06	CaHK10	7
CcRR9	CcLG06	CaRR25	7
CcRR8	CcLG06	CaRR5	6
CcRR16	CcLG08	CaRR31	3
CcHk6	CcLG09	CaHK13	4
CcHk6	CcLG09	CaHK5	4
CcRR3	Scaffold000011	CaRR9	5
CcHk18	Scaffold127746	CaHK12	Scaffold943
CcRR37	Scaffold128181	CaRR12	7
CcRR37	Scaffold128181	CaRR3	5
CcHk14	Scaffold128360	CaHK4	4
CcRR10	Scaffold129046	CaRR1	3
CcRR6	Scaffold134732	CaRR12	7

Medicago -Soybean			
Mt_TCS	Chr no.	GmTCS	Chr no.
MtHK3	chr1	Glyma.20G202200	20
MtHK6	chr1	Glyma.20G090000	20
MtHK3	chr1	Glyma.20G087000	20
MtHK4	chr1	Glyma.20G087000	20
Mt-HP1	chr1	Glyma.20G230800	20
MtHK5	chr1	Glyma.20G221800	20
MtHK4	chr1	Glyma.20G202200	20
MtHK5	chr1	Glyma.19G249100	19
Mt-HP1	chr1	Glyma.19G239100	19
MtHK6	chr1	Glyma.19G224200	19
Mt-HP2	chr1	Glyma.19G151900	19
MtHK1	chr1	Glyma.17G220800	17
MtHK1	chr1	Glyma.14G105400	14
Mt-HP2	chr1	Glyma.13G212800	13
MtRR6	chr1	Glyma.13G135900	13
MtRR6	chr1	Glyma.12G073900	12
MtHK4	chr1	Glyma.10G008500	10
MtRR6	chr1	Glyma.10G048100	10
MtHK4	chr1	Glyma.10G188500	10
MtHK5	chr1	Glyma.10G167300	10
Mt-HP1	chr1	Glyma.10G157800	10
MtHK6	chr1	Glyma.10G141400	10
MtHK3	chr1	Glyma.10G188500	10
MtHK3	chr1	Glyma.10G008500	10
Mt-HP2	chr1	Glyma.10G023200	10
MtRR4	chr1	Glyma.08G046800	8
Mt-HP2	chr1	Glyma.07G253100	7
MtRR4	chr1	Glyma.07G171200	7
MtHK1	chr1	Glyma.06G059100	6
MtHK1	chr1	Glyma.04G058400	4
MtHK5	chr1	Glyma.03G251700	3
Mt-HP1	chr1	Glyma.03G241700	3
MtHK6	chr1	Glyma.03G227300	3
MtHK7	chr1	Glyma.03G219100	3
Mt-HP2	chr1	Glyma.03G148600	3
Mt-HP2	chr1	Glyma.02G150800	2
MtRR4	chr1	Glyma.02G085900	2
MtRR7	chr2	Glyma.17G030600	17
Mt-HP4	chr2	Glyma.15G019500	15
Mt-HP6	chr2	Glyma.15G007400	15
MtHK10	chr2	Glyma.15G196500	15
MtRR7	chr2	Glyma.15G145200	15
MtHK9	chr2	Glyma.15G140000	15
Mt-HP3	chr2	Glyma.15G099800	15
Mt-HP3	chr2	Glyma.13G212800	13
Mt-HP4	chr2	Glyma.13G354700	13
MtHK10	chr2	Glyma.09G088500	9
MtRR7	chr2	Glyma.09G040000	9
MtHK9	chr2	Glyma.09G035500	9
Mt-HP6	chr2	Glyma.08G212800	8
Mt-HP4	chr2	Glyma.08G201100	8
Mt-HP6	chr2	Glyma.07G030100	7
Mt-HP4	chr2	Glyma.07G015600	7
MtRR7	chr2	Glyma.07G243300	7
Mt-HP3	chr2	Glyma.07G253100	7
Mt-HP3	chr2	Glyma.02G150800	2
MtRR17	chr3	Glyma.19G260400	19
MtRR15	chr3	Glyma.18G010700	18
MtRR11	chr3	Glyma.17G102200	17
MtHK13	chr3	Glyma.17G220800	17
MtRR17	chr3	Glyma.16G018000	16
MtHK13	chr3	Glyma.14G105400	14
MtRR15	chr3	Glyma.11G246400	11
MtHK12	chr3	Glyma.08G105000	8
MtRR15	chr3	Glyma.08G100900	8
MtRR17	chr3	Glyma.07G049400	7
MtRR11	chr3	Glyma.06G196200	6
MtHK13	chr3	Glyma.06G059100	6
MtRR17	chr3	Glyma.06G136600	6
MtHK12	chr3	Glyma.05G148100	5
MtRR15	chr3	Glyma.05G144500	5
MtRR17	chr3	Glyma.04G228300	4
MtRR11	chr3	Glyma.04G166300	4
MtHK13	chr3	Glyma.04G058400	4
MtRR17	chr3	Glyma.03G261300	3
MtRR31	chr4	Glyma.17G152800	17
MtRR33	chr4	Glyma.17G102200	17
MtRR34	chr4	Glyma.17G030600	17
MtRR34	chr4	Glyma.15G145200	15
Mt-HP7	chr4	Glyma.15G007400	15
MtRR29	chr4	Glyma.12G073900	12
MtRR31	chr4	Glyma.11G041300	11
MtRR34	chr4	Glyma.09G040000	9
MtHK14	chr4	Glyma.09G002600	9
Mt-HP7	chr4	Glyma.08G212800	8
MtRR34	chr4	Glyma.07G243300	7
Mt-HP7	chr4	Glyma.07G030100	7
MtRR33	chr4	Glyma.06G196200	6
MtRR31	chr4	Glyma.05G070200	5
MtRR33	chr4	Glyma.05G025000	5
MtRR33	chr4	Glyma.04G166300	4
MtRR31	chr4	Glyma.01G200800	1
MtRR39	chr5	Glyma.17G152800	17
MtHK15	chr5	Glyma.16G125100	16
MtHK16	chr5	Glyma.14G007100	14
MtHK15	chr5	Glyma.11G078300	11
MtRR39	chr5	Glyma.11G041300	11
MtRR39	chr5	Glyma.05G070200	5
MtHK15	chr5	Glyma.02G046600	2
MtHK16	chr5	Glyma.02G305900	2
MtRR39	chr5	Glyma.01G200800	1
MtHK15	chr5	Glyma.01G164800	1
MtHK18	chr7	Glyma.20G221800	20
Mt-HP8	chr7	Glyma.20G230800	20
MtHK18	chr7	Glyma.19G249100	19
MtRR54	chr7	Glyma.19G260400	19
Mt-HP8	chr7	Glyma.19G239100	19
MtHK17	chr7	Glyma.19G213300	19
MtRR52	chr7	Glyma.19G171300	19
MtRR50	chr7	Glyma.19G169700	19
MtRR54	chr7	Glyma.16G018000	16
MtHK18	chr7	Glyma.10G167300	10
Mt-HP8	chr7	Glyma.10G157800	10
MtRR54	chr7	Glyma.07G049400	7
MtRR54	chr7	Glyma.06G136600	6
MtRR54	chr7	Glyma.04G228300	4
MtHK18	chr7	Glyma.03G251700	3
MtRR54	chr7	Glyma.03G261300	3
Mt-HP8	chr7	Glyma.03G241700	3
MtHK17	chr7	Glyma.03G216700	3
MtRR52	chr7	Glyma.03G170100	3
MtRR56	chr8	Glyma.19G260400	19
MtRR60	chr8	Glyma.18G010700	18
MtHK19	chr8	Glyma.16G125100	16
MtRR56	chr8	Glyma.16G018000	16
MtHK19	chr8	Glyma.11G078300	11
MtRR60	chr8	Glyma.11G246400	11
MtHK20	chr8	Glyma.08G105000	8
MtRR60	chr8	Glyma.08G100900	8
MtHK21	chr8	Glyma.08G049000	8
MtHK21	chr8	Glyma.07G173700	7
MtRR57	chr8	Glyma.07G079000	7
MtRR56	chr8	Glyma.07G049400	7
MtRR56	chr8	Glyma.06G136600	6
MtHK21	chr8	Glyma.05G241600	5
MtHK20	chr8	Glyma.05G148100	5
MtRR60	chr8	Glyma.05G144500	5
MtRR56	chr8	Glyma.04G228300	4
MtRR56	chr8	Glyma.03G261300	3
MtRR57	chr8	Glyma.03G017700	3
MtHK21	chr8	Glyma.02G087000	2
MtHK19	chr8	Glyma.02G046600	2
MtHK19	chr8	Glyma.01G164800	1

C. cajan-Medicago			
CcTCS	Chr no.	MtTCS	Chr no.
CcRR23	CcLG01	MtRR57	chr8
CcHk19	CcLG02	MtHK7	chr1
CcHk11	CcLG03	MtHK6	chr1
CcRR29	CcLG03	MtRR11	chr3
CcHk10	CcLG03	MtHK13	chr3
CcRR34	CcLG03	MtRR17	chr3
CcRR34	CcLG03	MtRR54	chr7
CcRR31	CcLG03	MtRR52	chr7
CcRR34	CcLG03	MtRR56	chr8
CcRR20	CcLG04	MtRR6	chr1
CcRR20	CcLG04	MtRR29	chr4
CcRR9	CcLG06	MtRR31	chr4
CcRR9	CcLG06	MtRR39	chr5
CcHk3	CcLG06	MtHK15	chr5
CcHk3	CcLG06	MtHK19	chr8
CcRR16	CcLG08	MtRR51	chr7
CcHk14	Scaffold128360	MtHK1	chr1
CcHk12	Scaffold129714	MtHK9	chr2

C. cajan -Soybean			
CcTCS	Chr no.	GmTCS	Chr no.
CcRR21	CcLG01	Glyma.08G100900	8
CcHP9	CcLG02	Glyma.20G230800	20
CcHP8	CcLG02	Glyma.19G151900	19
CcHP9	CcLG02	Glyma.19G239100	19
CcHP8	CcLG02	Glyma.15G099800	15
CcHP8	CcLG02	Glyma.13G212800	13
CcHP9	CcLG02	Glyma.10G157800	10
CcHP8	CcLG02	Glyma.07G253100	7
CcHP9	CcLG02	Glyma.03G241700	3
CcHP8	CcLG02	Glyma.02G150800	2
CcHP2	CcLG03	Glyma.19G151900	19
CcHP2	CcLG03	Glyma.15G099800	15
CcHP2	CcLG03	Glyma.13G212800	13
CcHP2	CcLG03	Glyma.10G023200	10
CcHP2	CcLG03	Glyma.02G150800	2
CcHP7	CcLG11	Glyma.15G099800	15
CcHP7	CcLG11	Glyma.13G212800	13
CcHP7	CcLG11	Glyma.07G253100	7
CcHP1	Scaffold125995	Glyma.19G239100	19
CcHP1	Scaffold125995	Glyma.03G241700	3
CcHP10	Scaffold133616	Glyma.06G088100	6
CcRR23	CcLG01	Glyma.07G079000	7
CcRR21	CcLG01	Glyma.05G144500	5
CcRR23	CcLG01	Glyma.03G017700	3
CcHk11	CcLG03	Glyma.20G090000	20
CcHk11	CcLG03	Glyma.19G224200	19
CcRR31	CcLG03	Glyma.19G171300	19
CcRR34	CcLG03	Glyma.19G260400	19
CcRR33	CcLG03	Glyma.19G250500	19
CcRR30	CcLG03	Glyma.17G217100	17
CcRR34	CcLG03	Glyma.16G018000	16
CcRR30	CcLG03	Glyma.14G110600	14
CcHk11	CcLG03	Glyma.10G141400	10
CcRR34	CcLG03	Glyma.07G049400	7
CcHk10	CcLG03	Glyma.06G059100	6
CcRR30	CcLG03	Glyma.06G063500	6
CcRR29	CcLG03	Glyma.06G196200	6
CcRR34	CcLG03	Glyma.06G136600	6
CcRR34	CcLG03	Glyma.04G228300	4
CcHk10	CcLG03	Glyma.04G058400	4
CcRR30	CcLG03	Glyma.04G062500	4
CcHk11	CcLG03	Glyma.03G227300	3
CcRR31	CcLG03	Glyma.03G170100	3
CcRR34	CcLG03	Glyma.03G261300	3
CcRR33	CcLG03	Glyma.03G252900	3
CcRR20	CcLG04	Glyma.U034500	U034500
CcRR20	CcLG04	Glyma.13G135900	13
CcRR20	CcLG04	Glyma.12G073900	12
CcRR20	CcLG04	Glyma.10G048100	10
CcRR8	CcLG06	Glyma.18G130900	18
CcRR9	CcLG06	Glyma.17G152800	17
CcHk3	CcLG06	Glyma.16G125100	16
CcHk3	CcLG06	Glyma.11G078300	11
CcRR9	CcLG06	Glyma.11G041300	11
CcRR8	CcLG06	Glyma.08G292400	8
CcRR9	CcLG06	Glyma.05G070200	5
CcHk3	CcLG06	Glyma.02G046600	2
CcRR8	CcLG06	Glyma.02G027400	2
CcHk3	CcLG06	Glyma.01G164800	1
CcRR9	CcLG06	Glyma.01G200800	1
CcRR8	CcLG06	Glyma.01G037500	1
CcRR16	CcLG08	Glyma.19G169700	19
CcRR15	CcLG08	Glyma.08G046700	8
CcHk7	CcLG08	Glyma.08G049000	8
CcHk7	CcLG08	Glyma.07G173700	7
CcHk7	CcLG08	Glyma.05G241600	5
CcHk7	CcLG08	Glyma.02G087000	2
CcHk6	CcLG09	Glyma.10G008500	10
CcRR3	Scaffold000011	Glyma.06G142300	6
CcHk18	Scaffold127746	Glyma.19G213300	19
CcHk18	Scaffold127746	Glyma.03G216700	3
CcRR37	Scaffold128181	Glyma.06G187000	6
CcRR37	Scaffold128181	Glyma.05G033000	5
CcRR37	Scaffold128181	Glyma.04G177900	4
CcHk14	Scaffold128360	Glyma.17G220800	17
CcHk14	Scaffold128360	Glyma.14G105400	14
CcHk14	Scaffold128360	Glyma.06G059100	6
CcHk14	Scaffold128360	Glyma.04G058400	4
CcRR10	Scaffold129046	Glyma.19G132300	19
CcRR10	Scaffold129046	Glyma.03G130000	3
CcHk12	Scaffold129714	Glyma.15G140000	15
CcHk12	Scaffold129714	Glyma.09G035500	9
CcHk5	Scaffold132418	Glyma.07G173700	7
CcHk5	Scaffold132418	Glyma.02G087000	2
CcRR24	Scaffold132616	Glyma.09G040000	9
CcRR18	Scaffold133013	Glyma.13G077500	13
CcRR18	Scaffold133013	Glyma.06G114900	6
CcRR18	Scaffold133013	Glyma.04G247800	4
CcRR6	Scaffold134732	Glyma.17G093900	17
CcRR6	Scaffold134732	Glyma.06G187000	6
CcRR6	Scaffold134732	Glyma.05G033000	5
CcHk13	Scaffold135298	Glyma.14G007100	14
CcHk13	Scaffold135298	Glyma.02G305900	2
CcRR1	Scaffold136490	Glyma.11G246400	11
CcRR1	Scaffold136490	Glyma.08G100900	8
CcHk1	Scaffold136490	Glyma.08G105000	8
CcRR1	Scaffold136490	Glyma.05G144500	5
CcHk1	Scaffold136490	Glyma.05G148100	5

Supplemental Table 5: A list of orthologous pairs in legumes with Arabidopsis

Ca_TCS	At_TCS	Ka	Ks	Ka/Ks
CaHK1	AT1G09570	0.1793	1.8228	0.098365153
CaHK10	AT2G17820	0.2659	2.5319	0.105019945
CaHK13	AT1G04310	0.4502	2.6286	0.171269878
CaHK14	AT5G35750	0.3246	1.8005	0.180283255
CaHK15	AT2G47430	0.4869	2.4451	0.19913296
CaHK16	AT1G09570	0.1329	2.1955	0.060532908
CaHK18	AT1G27320	0.2152	1.7702	0.121568184
CaHK19	AT2G01830	0.2111	2.6824	0.078698181
CaHK3	AT2G17820	0.2564	1.7399	0.147364791
CaHK4	AT5G10680	0.4402	2.4218	0.181765629
CaHK5	AT3G23150	0.2727	3.8381	0.07105078
CaHK5	AT1G04310	0.388	2.6886	0.144313025
CaHK6	AT3G04580	0.2835	1.6089	0.176207347
CaHK7	AT1G27320	0.2108	2.0551	0.102574084
CaHK8	AT5G10680	0.5131	2.3445	0.218852634
CaPRR3	AT2G46790	0.5869	2.0003	0.293405989
CaPRR4	AT5G61380	0.3448	2.046	0.168523949
CaPRR5	AT5G02810	0.5152	3.2443	0.15880159
CaPRR5	AT5G60100	0.5645	2.203	0.256241489
CaRR11	AT1G68210	0.87	1.3104	0.663919414

Mt_TCS	At_TCS	Ka	Ks	Ka/Ks
MtHK1	AT5G10680	0.436	1.8211	0.239415738
MtHK12	AT1G27320	0.2195	2.1254	0.103274678
MtHK13	AT5G10680	0.584	0	
MtHK15	AT2G17820	0.2597	1.6712	0.155397319
MtHK19	AT2G17820	0.2679	2.6509	0.101060017
MtHK20	AT1G27320	0.2106	2.1406	0.098383631
MtHK21	AT2G01830	0.2076	2.1336	0.097300337
MtHK3	AT3G23150	0.338	2.3487	0.143909397
MtHK3	AT1G04310	0.4136	0	
MtHK4	AT3G23150	0.2834	3.0562	0.092729533
MtHK4	AT1G04310	0.3969	2.6812	0.148030733
MtHK5	AT3G04580	0.2931	1.5412	0.190176486
MtHK6	AT1G09570	0.1334	2.5763	0.051779684
MtHK7	AT2G47430	0.6035	2.8047	0.215174528
Mt-HP2	AT1G03430	0.3648	0	
Mt-HP4	AT3G16360	0.2156	1.2793	0.168529665
Mt-HP6	AT1G80100	0.1472	0	
MtRR17	AT2G46790	0.5188	2.4481	0.211919448
MtRR29	AT5G60100	0.4033	1.604	0.251433915
MtRR33	AT5G61380	0.3957	1.7082	0.231647348
MtRR54	AT4G00760	1.1118	2.9816	0.372887041
MtRR54	AT2G46790	0.5403	2.5478	0.212065311
MtRR56	AT4G00760	1.0004	2.7112	0.368987902
MtRR56	AT2G46790	0.5758	2.3597	0.24401407
MtRR6	AT5G60100	0.4626	2.4443	0.189256638
MtRR6	AT5G02810	0.377	2.0548	0.183472844

Cc_TCS	At_TCS	Ka	Ks	Ka/Ks
CcHk1	AT1G27320	0.2017	2.0543	0.098184296
CcHk20	AT3G04580	0.272	0	
CcHk3	AT2G17820	0.225	1.8126	0.124131082
CcHk6	AT3G23150	0.2627	0	
CcHk7	AT2G01830	0.2141	2.1728	0.098536451
CcHP2	AT1G03430	0.3769	0	
CcRR16	AT3G04280	0.7306	3.036	0.240645586
CcRR20	AT5G02810	0.4791	2.5736	0.186159465
CcRR20	AT5G60100	0.4062	1.8963	0.214206613
CcRR23	AT4G00760	1.0073	1.7352	0.580509451
CcRR34	AT4G00760	1.0425	1.5547	0.670547372
CcRR34	AT2G46790	0.5414	1.9751	0.274112703
CcRR9	AT4G18020	0.4329	3.3488	0.129270186

Supplemental Table 6: The ratio of total genes and TCS gene in *Medicago*-*C. cajan*, *Medicago*-chickpea and chickpea-*C. cajan*

Organism	Total number of genes
**Chickpea (Ca)**	50894
***Medicago* (Mt)**	40071
***C. cajan* (Cc)**	28269

	Gene Ratio	TCS Ratio
**Mt/Cc**	1.27	1.28
**Mt/Ca**	1.8	1.43
**Cc/Ca**	1.41	1.11
